# A core-shell Au@Cu_2-x_Se heterogeneous metal nanocomposite for photoacoustic and computed tomography dual-imaging-guided photothermal boosted chemodynamic therapy

**DOI:** 10.1186/s12951-021-01159-x

**Published:** 2021-12-07

**Authors:** Le Zhang, Chunjuan Jiang, Bing Li, Zhengwang Liu, Bingxin Gu, Simin He, Panli Li, Yun Sun, Shaoli Song

**Affiliations:** 1 Department of Nuclear Medicine, Shanghai Proton and Heavy Ion Center, Fudan University Cancer Hospital, Shanghai, 201321 China; 2 Department of Research and Development, Shanghai Proton and Heavy Ion Center, Shanghai, 201321 China; 3 Shanghai Key Laboratory of Radiation Oncology (20dz2261000), Shanghai, China; 4 Shanghai Engineering Research Center of Proton and Heavy Ion Radiation Therapy, Shanghai, China

**Keywords:** Chemodynamic therapy, Photothermal therapy, Core-shell metal nanoparticles, Fenton-like reaction, Tumor imaging

## Abstract

Chemodynamic therapy (CDT) has aroused extensive attention for conquering cancers because of its high specificity and low invasiveness. Quick generation of hydroxyl radicals (**·**OH) during CDT could induce more irreparable damage to cancer cells. The generation rate of **·**OH could be magnified via the selection of suitable nanocatalysts or under the assistance of exogenous thermal energy from photothermal therapy (PTT). Here, we construct a kind of monodisperse core-shell Au@Cu_2-x_Se heterogeneous metal nanoparticles (NPs) for PTT boosted CDT synergistic therapy. Due to the localized surface plasmon resonance (LSPR) coupling effect in the core-shell structure, the photothermal conversion efficiency of Au@Cu_2-x_Se NPs is up to 56.6%. The in situ generated heat from photothermal can then accelerate the Fenton-like reaction at Cu^+^ sites to produce abundant **·**OH, which will induce apoptotic cell death by attacking DNA, contributing to a heat-boosted CDT. Both in vitro and in vivo results showed that after this synergistic therapy, tumors could be remarkably suppressed. Guided by photoacoustic (PA) and computed tomography (CT) imaging, the therapeutic effects were more specified. Our results revealed that PA and CT dual-imaging-guided PTT boosted CDT synergistic therapy based on core-shell Au@Cu_2-x_Se NPs is an effective cancer treatment strategy.

**Supplementary Information:**

The online version contains supplementary material available at 10.1186/s12951-021-01159-x.

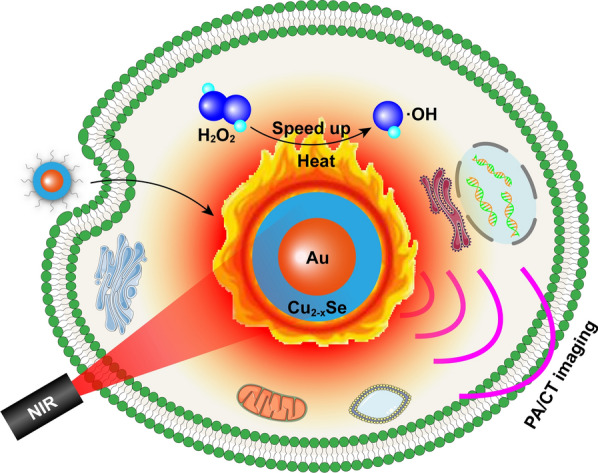

## Introduction

Reactive oxygen species (ROS), mainly including hydrogen peroxide (H_2_O_2_), singlet oxygen (^1^O_2_), superoxide anion (O_2_^·-^), and hydroxyl radical (**·**OH), have significant influences on various physiological functions of cancer cells. At moderate levels, ROS could facilitate cancer occurrence and development either by inducing the mutation of genomic DNA or by acting as pro-oncogenic signaling molecules. At high levels, ROS can cause severe damage and death of cancer cells via attacking cellular components such as lipids, proteins, and DNA [1–3]. Therefore, upregulating the ROS levels in cancer cells represents a promising strategy for cancer therapy. Among various ROS-enhanced anticancer strategies, chemodynamic therapy (CDT), which uses Fenton or Fenton-like reaction catalyzed by nanomaterials to decompose less-reactive H_2_O_2_ overexpressed in tumor cells into highly cytotoxic **·**OH, is an emerging therapeutic route because of the merits of high therapeutic specificity and low invasiveness [4, 5]. However, the sluggish Fenton kinetics result in the unsatisfactory efficacy of CDT, because the generation rate of **·**OH is not quick enough to overcome the intracellular antioxidant system. Therefore, how to speed up the sluggish Fenton kinetics is a major concern for CDT at present.

Nanocatalysts play an irreplaceable role in CDT. Fe^2+^-based nanomaterials are the widely exploited nanocatalysts for CDT, such as Fe_3_O_4_ nanoparticles [6], Fe_2_P nanorods [7], and FePS_3_ nanosheets [8]. However, Fe^2+^-based nanomaterials only exhibit high Fenton catalytic activity in low pH conditions (pH 2−4). The weakly acid tumor microenvironment (TME, pH ~6.5) is not conducive to Fe^2+^-based nanomaterials [9]. It has been reported that Cu^+^-catalyzed Fenton-like reactions are easier to occur in weakly acidic and neutral media, compared with Fe^2+^-catalyzed Fenton reactions [9]. So Cu^+^-containing catalysts may be a better candidate.

Stimulation by external energy fields, such as heat, light, and ultrasound, is more workable to speed up Fenton kinetics to improve CDT effects [4, 5]. Photothermal therapy (PTT) based on nanomaterials is another effective but less invasive therapeutic alternative, which converts near-infrared (NIR) light into local heat to realize tumor ablation. The hyperthermia generated in PTT can not only kill cancer cells but also speed up the Fenton-like reaction in CDT, consequently achieving a synergistic therapeutic outcome [8, 10, 11]. It has been reported that by constructing Au@semiconductor core-shell dual plasmonic hybrid nanocomposite, the photothermal conversion efficiency of nanocomposite could be efficiently improved because the core-shell structure could couple the localized surface plasmon resonance (LSPR) of two components to a maximum degree [12–14]. So introducing Au into Cu^+^-based nanomaterials can simultaneously achieve PTT and CDT in a single platform. Besides, both photoacoustic (PA) imaging and PTT depend on a similar NIR absorption mechanism, plus Au has computed tomography (CT) imaging property due to its X-ray attenuation capability, so PA/CT dual-imaging guided PTT + CDT synergistic therapy will be realized.

**Scheme 1:**
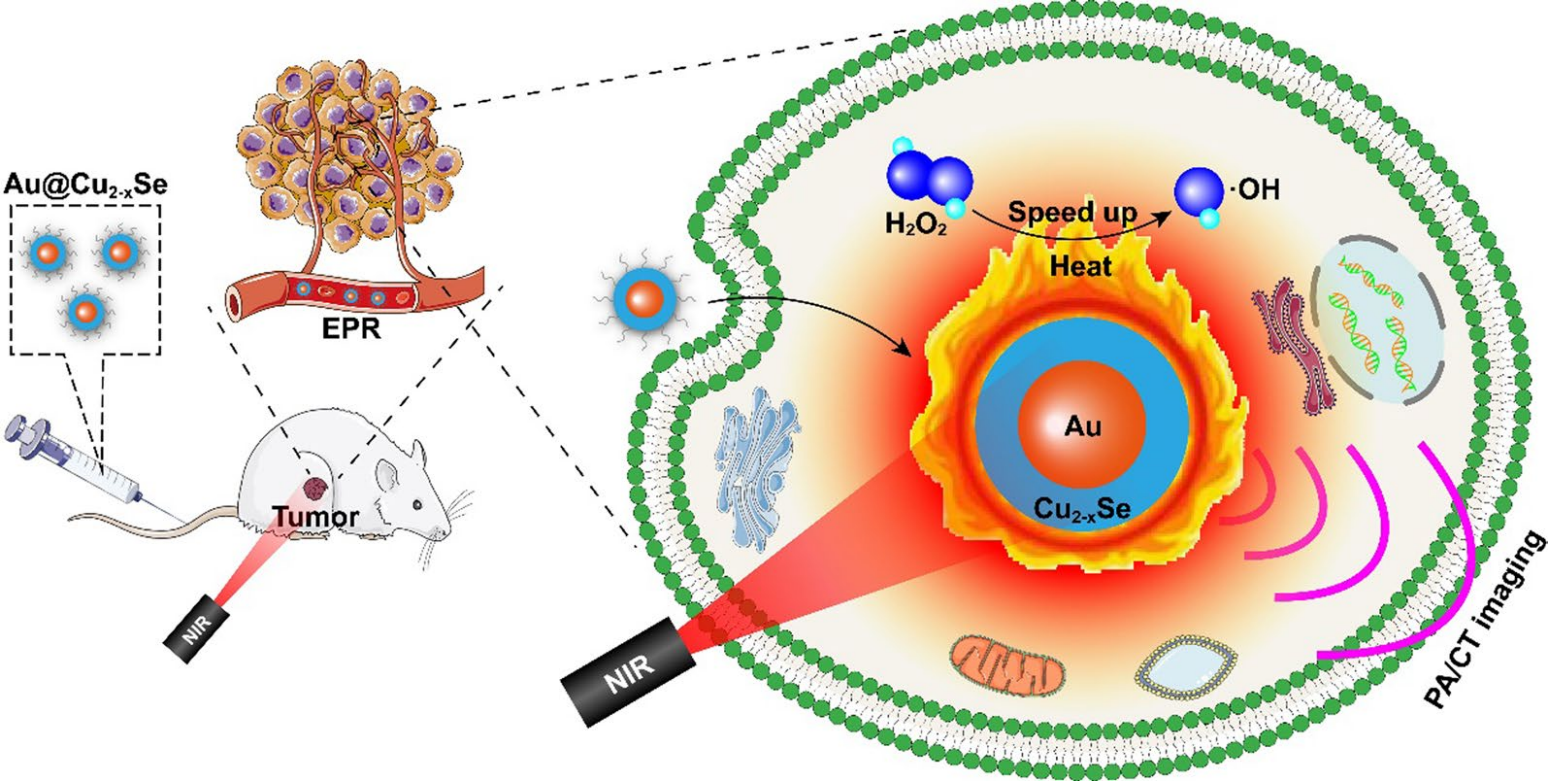
Schematic illustration of core-shell Au@Cu_2-x_Se NPs for PA/CT imaging-guided PTT + CDT synergistic cancer therapy

Herein, we developed a kind of monodisperse core-shell Au@Cu_2-x_Se hybrid metal nanoparticles (NPs) as a multifunctional theranostic nanoplatform for PA/CT dual-imaging-guided combinational tumor therapy of PTT and the boosted CDT (Scheme 1). The core-shell structure strengthens the LSPR coupling between Au and Cu_2-x_Se and thus heightens the overall photothermal conversion efficiency of Au@Cu_2-x_Se NPs. The existence of Cu^+^ enables high Fenton-like catalytic activity of Au@Cu_2-x_Se NPs for CDT. More importantly, the Fenton-like reaction at Cu^+^ sites can be accelerated by the in situ generated heat from PTT, wherein the produced ·OH will cause DNA breaks and then initiate cell apoptosis. Finally, the quick hyperthermia by PTT and quick production of ·OH by the boosted CDT defeat the intracellular antioxidant systems and induce irreversible damage to tumor cells. In addition, Au@Cu_2-x_Se NPs have the potential as a contrast agent for PA and CT imaging, which could make this combinational therapy more specified. The heterogeneous metal nanocomposite paves the way of precisely controlling the reactive oxygen microenvironment in tumor to inhibit tumor progression.

## Results and discussion

### Synthesis and characterization of Au@Cu_2-x_Se NPs

**Fig. 1:**
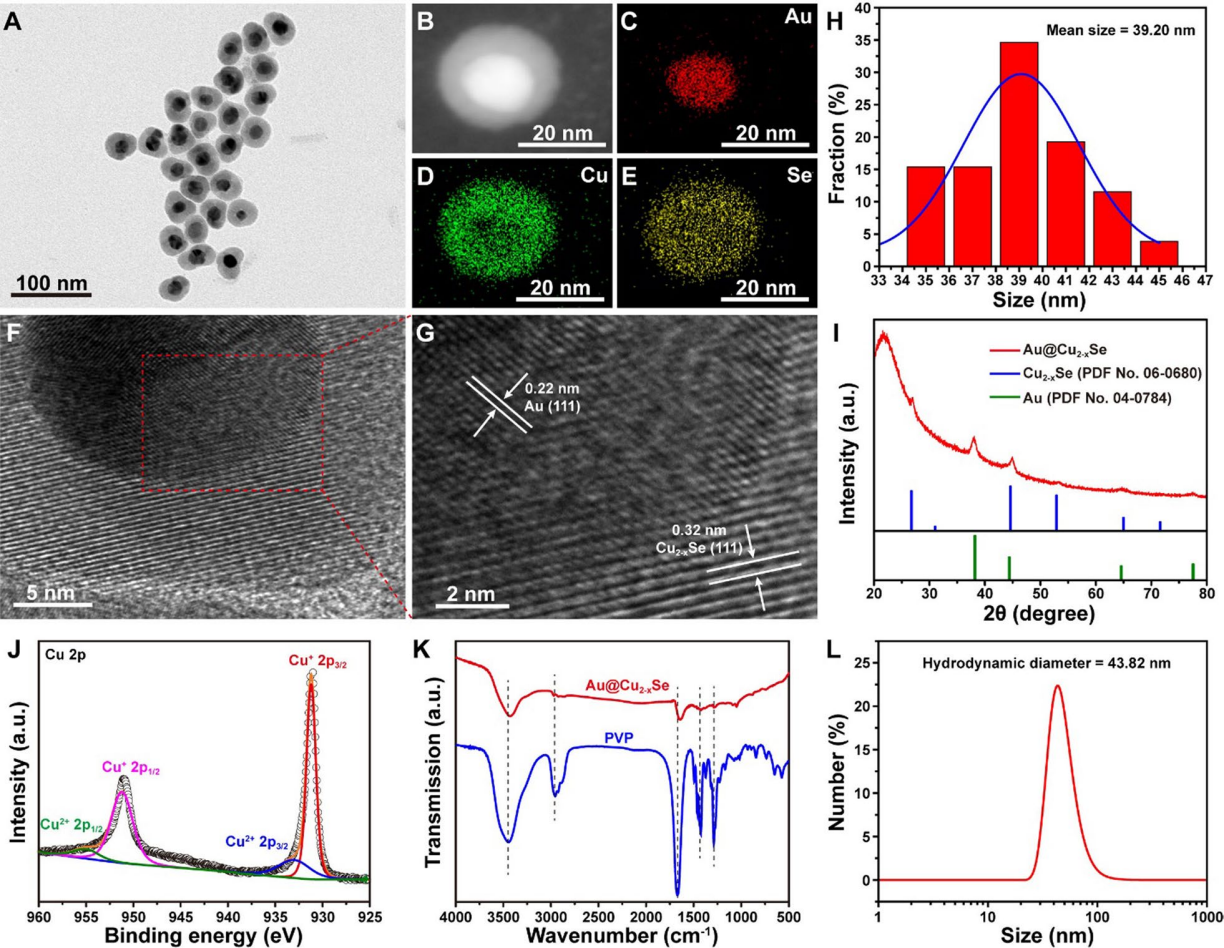
** A** TEM image, **B–E** elemental mapping images, and **F**, **G** HRTEM images of Au@Cu_2-x_Se NPs. **H** Size distribution of Au@Cu_2-x_Se NPs counted from A. **I** XRD pattern of Au@Cu_2-x_Se NPs. **J** Cu 2p XPS spectrum of Au@Cu_2-x_Se NPs. **K** FTIR spectra of Au@Cu_2-x_Se NPs and pure PVP. **L** Hydrodynamic size of Au@Cu_2-x_Se NPs dispersed in water (100 µg mL^−1^)

Monodisperse core-shell Au@Cu_2-x_Se NPs were prepared via an eco-friendly two-step procedure. Au cores were first obtained by reducing HAuCl_4_ with sodium citrate. Subsequently, Cu_2-x_Se grew onto the Au surface by co-reducing Cu^2+^ and SeO_2_ with ascorbic acid (Additional file 1: Fig. S1) under the assistance of polyvinylpyrrolidone (PVP). Transmission electron microscope (TEM) image (Fig. 1A, H) demonstrates the core-shell structure of Au@Cu_2-x_Se with an average size of 39.20 nm. Corresponding element mapping (Fig. 1B–E) confirms that Cu and Se are uniformly distributed around the Au core. The measured lattice distances of core and shell from high-resolution TEM image (HRTEM, Fig. 1F, G) are 0.22 and 0.32 nm, which correspond to the (111) plane of Au and (111) plane of Cu_2-x_Se [15, 16], respectively. The selected area electron diffraction (SAED) pattern (Additional file 1: Fig. S2) reveals that Au@Cu_2-x_Se NPs are highly crystalline. X-ray diffraction (XRD) pattern (Fig. 1I) proves the formation of cubic phase Au (PDF No. 04-0784) and cubic berzelianite phase Cu_2-x_Se (PDF No. 06-0680). X-ray photoelectron spectroscopy (XPS) was then performed to analyze the chemical compositions of Au@Cu_2-x_Se. In the high-resolution Cu 2p XPS spectra (Fig. 1J), the peaks at 931.2 and 951.2 eV can be attributed to the Cu^+^ state, and the peaks at 933.0 and 955.0 eV is related to the Cu^2+^ state [16]. The peak area of Cu^+^ is larger than that of Cu^2+^, indicating that Cu mainly exists as Cu^+^. This ensures the good catalytic activity of Au@Cu_2-x_Se for Fenton-like reaction because Cu^+^ has better activity than Cu^2+^ [9, 17]. N 1 s XPS spectrum (Additional file 1: Fig. S3) implies the coating of PVP on Au@Cu_2-x_Se surface. This can be further substantiated by Fourier transform infrared (FTIR) spectra (Fig. 1K), wherein Au@Cu_2-x_Se presents the four characteristic absorption bands of PVP at 1289, 1426, 1664, and 2957 cm^−1^, respectively [18, 19]. The modification with PVP endows Au@Cu_2-x_Se proper zeta potential (− 24.51 mV, Additional file 1: Fig. S4) and good biocompatibility in physiological conditions. The hydrodynamic size of Au@Cu_2-x_Se determined by dynamic light scattering (DLS) is around 43.82 nm (Fig. 1L), which is suitable for biomedical applications. Besides, the morphology of Au@Cu_2-x_Se NPs after 14-day incubation either in saline or in RPMI-1640 complete medium has no obvious changes (Additional file 1: Fig. S5), indicating the excellent stability of Au@Cu_2-x_Se NPs for durable therapy.

### Photothermal performance of Au@Cu_2-x_Se NPs

**Fig. 2:**
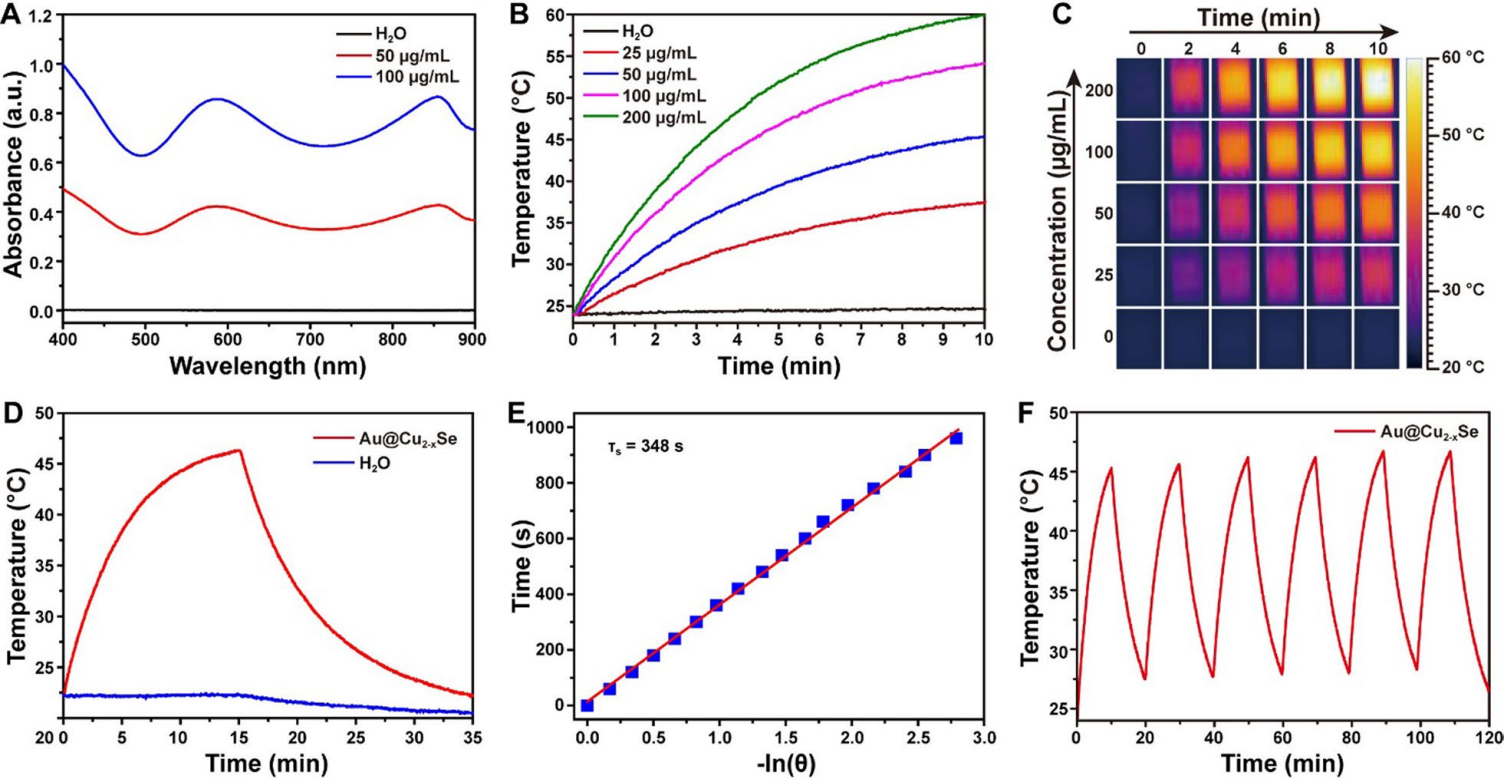
** A** UV-vis-NIR absorption spectra of Au@Cu_2-x_Se aqueous suspensions. **B** Photothermal curves and **C** corresponding thermal photographs of Au@Cu_2-x_Se aqueous suspensions with different concentrations under NIR irradiation. **D** Heating-cooling curves of Au@Cu_2-x_Se aqueous suspension (50 µg mL^−1^) and pure water irradiated with NIR for 15 min and then naturally cooled to room temperature. **E** The calculation of time constant (*τ*_s_) for the heat transfer from system obtained by applying the linear time data from the cooling period versus the negative natural logarithm of driving force temperature (*θ*). **F** Photothermal stability curve of Au@Cu_2-x_Se aqueous suspension (50 µg mL^−1^). The power density of 808 nm NIR was maintained at 1.0 W cm^−2^ in these photothermal tests

Ultraviolet-visible-infrared (UV-vis-NIR) absorption spectra (Fig. 2A) revealed that Au@Cu_2-x_Se NPs aqueous suspensions possessed strong absorption in NIR region, implying the potential as a nanoagent for PTT. To investigate the detailed photothermal performance, the Au@Cu_2-x_Se NPs aqueous suspensions with different concentrations were exposed to an 808 nm NIR laser at a power density of 1.0 W cm^−2^. Significant concentration-dependent temperature increases of solutions were observed under laser irradiation (Fig. 2B, C). For example, 10 min NIR irradiation for 50 µg mL^−1^ solution induced a quick heat from room temperature to 45 °C. In contrast, the temperature of pure water had negligible change under identical laser condition. The calculated photothermal conversion efficiency of Au@Cu_2-x_Se NPs was about 56.6% (Fig. 2D, E), which is much superior to most recently reported materials, such as Au-Fe_2_C nanoparticles (30.2%) [20], PVP-Bi nanodots (30%) [21], V_2_C nanosheets (48%) [22], NbSe_2_ nanosheets (42.9%) [23] (see details in Additional file 1: Table S1). To evaluate the photothermal stability of Au@Cu_2-x_Se NPs, the temperature variation of Au@Cu_2-x_Se NPs dispersion was recorded under six cycles of heating and cooling process upon NIR laser exposure. There was little deterioration of photothermal performance during each cycle (Fig. 2F), illustrating that Au@Cu_2-x_Se NPs have a durable therapeutic efficiency for PTT.

To identify the role of core-shell structure in the photothermal conversion, the photothermal performance of Au@Cu_2-x_Se NPs was compared with that of individual Au NPs and individual Cu_2-x_Se NPs. TEM characterizations confirm the successful preparation of Au NPs (Additional file 1: Fig. S6) and Cu_2-x_Se NPs (Additional file 1: Fig. S7). As shown in Additional file 1: Fig. S8, the absorbance of Au@Cu_2-x_Se NPs at 808 nm was higher than that of Au NPs, Cu_2-x_Se NPs, and physical mixture of Au NPs and Cu_2-x_Se NPs (Additional file 1: Fig. S8A, C). The temperature increase rate of Au@Cu_2-x_Se NPs dispersion was also faster than that of Au NPs, Cu_2-x_Se NPs, and physical mixture of Au NPs and Cu_2-x_Se NPs dispersion (Additional file 1: Fig. S8B, D). Based on these results and combined with previous reports, such high photothermal property of Au@Cu_2-x_Se NPs can be attributed to its unique core-shell structure. Combining the Au metal and Cu_2-x_Se semiconductor into one core-shell hybrid nanocomposite could achieve a strong LSPR coupling between Au and Cu_2-x_Se and thus enhance the NIR absorption, consequently leading to the improved photothermal effect [12–14].

### Photothermal enhanced Fenton-like catalytic activity of Au@Cu_2-x_Se NPs

**Fig. 3:**
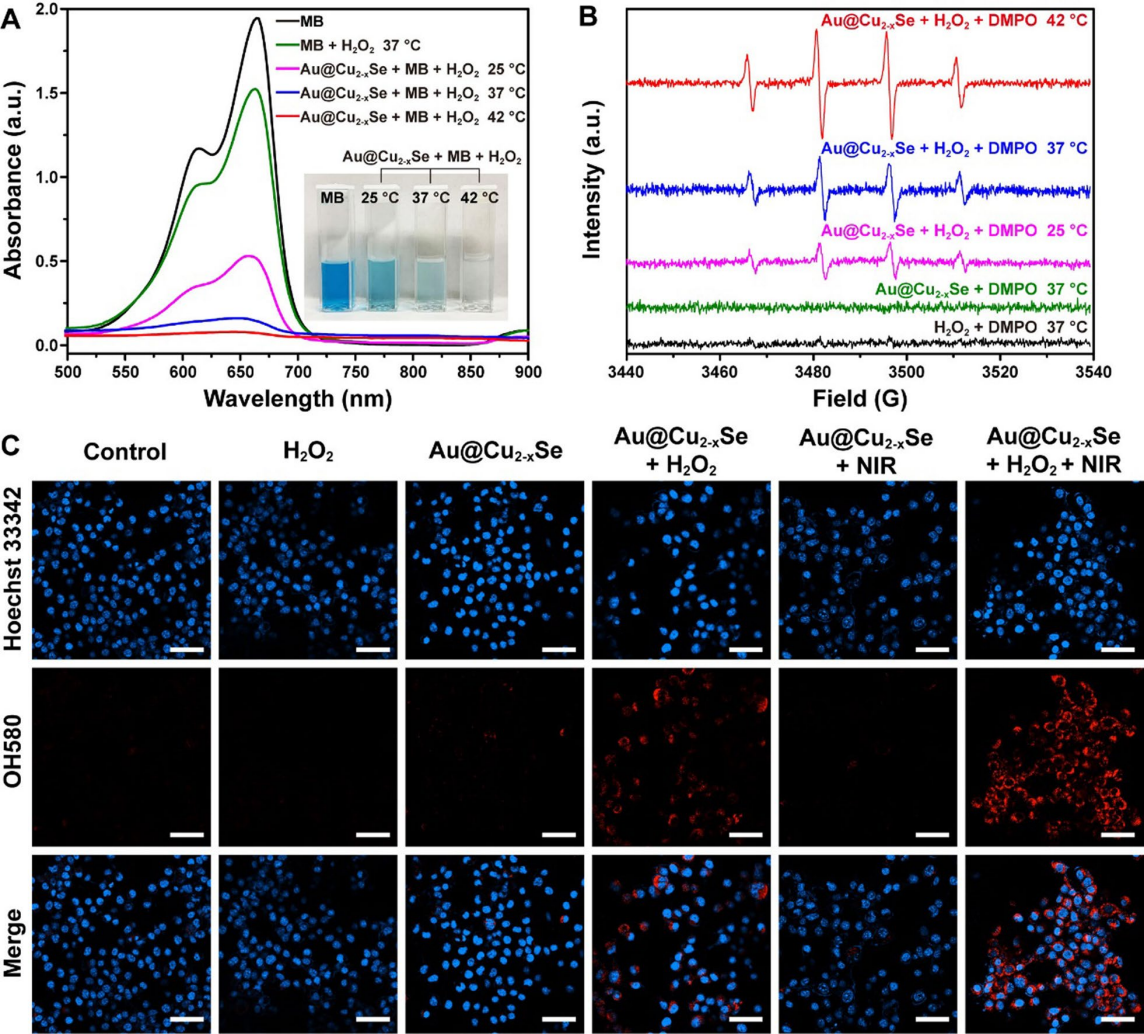
** A** UV-vis-NIR absorption spectra of MB aqueous solutions containing H_2_O_2_ and Au@Cu_2−x_Se NPs after treatments at different temperatures. The inset photograph shows the corresponding color changes of MB solutions. Au@Cu_2−x_Se: 100 µg mL^−1^. MB: 10 µg mL^−1^. H_2_O_2_: 10 mM. **B** ESR spectra of Au@Cu_2−x_Se NPs recorded at different temperatures. The spin trap was DMPO. Au@Cu_2−x_Se: 50 µg mL^−1^. DMPO: 50 mM. H_2_O_2_: 100 µM. **C** CLSM images of 4T1 tumor cells after different treatments, where Hoechst 33,342 (blue) and OH580 (red) were used to observe the cell nucleus and ·OH generation, respectively. Scale bar = 50 μm. Au@Cu_2−x_Se: 50 µg mL^−1^. H_2_O_2_: 100 µM. NIR: 808 nm laser, 1.0 W cm^−2^ for 5 min

The therapeutic efficiency of CDT depends on the generation rate of ·OH in the Fenton-like reaction. The existence of Cu^+^ theoretically proves that Au@Cu_2-x_Se NPs have Fenton-like activity [9, 17]. Degradation experiments of methylene blue (MB) were then carried out to evaluate the heat-enhanced Fenton-like activity of Au@Cu_2-x_Se NPs. After incubating MB with Au@Cu_2-x_Se NPs at 25, 37, or 42 °C for 30 min in water containing H_2_O_2_, the absorbance of supernatant was measured, where the absorbance decrease at 664 nm was induced by ·OH radical. The temperature-dependent MB degradation is shown in Fig. 3A. A slight decrease of absorbance in MB + H_2_O_2_ group was observed, implying that H_2_O_2_ might degrade part of MB. More significant decrease of absorbance in Au@Cu_2-x_Se + MB + H_2_O_2_ groups was observed, and the extent of decrease increased with the increase of temperature. The absorbance at 42 °C was 7.43 times lower than that at 25 °C. These results manifest that thermal energy could indeed accelerate ·OH production. A similar temperature-dependent MB degradation phenomenon was also observed on pure Cu_2-x_Se NPs (Additional file 1: Fig. S9), indicating that the Fenton-like catalytic activity of Au@Cu_2-x_Se NPs derives from Cu_2-x_Se shell. Of course, MB degradation could also be achieved by prolonging the reaction time (Additional file 1: Fig. S10), but this is not the optimal strategy.

The generation of ·OH at different temperatures was then detected via electron spin resonance (ESR) by using 5,5-dimethyl-1-pyrroline N-oxide (DMPO) as a spin trap. DMPO captures short-lived ·OH radicals to form relatively long-lived DMPO-OH adducts, which exhibit typical four peaks with relative intensities of 1:2:2:1 in ESR spectrum [24, 25]. As shown in Fig. 3B, compared with H_2_O_2_ or Au@Cu_2-x_Se alone, Au@Cu_2-x_Se + H_2_O_2_ group exhibited higher ESR signal and DMPO-OH characteristic peaks. Meanwhile, higher characteristic signal peaks could be recognized at higher temperatures, supporting the enhancing effect of thermal energy toward Fenton-like reaction catalyzed by Au@Cu_2-x_Se NPs.

Based on above results, the photothermal accelerated ·OH generation in Fenton-like reaction catalyzed by Au@Cu_2-x_Se NPs was verified. As expected, under 10 min of NIR irradiation at 1.0 W cm^−2^, the temperature of mixed solution of Au@Cu_2-x_Se NPs (100 µg mL^−1^), MB, and H_2_O_2_ increased from room temperature to 58 °C (Additional file 1: Fig. S11A). After removing Au@Cu_2-x_Se NPs, the supernatant became colorless, and its absorbance was reduced by 99% (Additional file 1: Fig. S11B, C). This result further validates the feasibility of using photothermal to enhance CDT based on Au@Cu_2-x_Se NPs.

### In vitro photothermal and chemodynamic therapy based on Au@Cu_2-x_Se NPs

Before executing the anticancer effect of Au@Cu_2-x_Se NPs via PTT + CDT in vitro, confocal laser scanning microscope (CLSM) was adopted to show the production of ·OH in 4T1 tumor cells. First, 2′,7′- dichlorofluorescein diacetate (DCFH-DA) was used to detect the ROS in cells. When DCFH-DA freely passes through cell membrane, intracellular esterase will hydrolyze it to produce DCFH without fluorescence, which then is oxidized by intracellular ROS to form DCF with green fluorescence. As expected, Au@Cu_2-x_Se + H_2_O_2_ group exhibited a green fluorescence in CLSM images, whereas Au@Cu_2-x_Se + H_2_O_2_ + NIR group showed the strongest green fluorescence (Additional file 1: Fig. S12), manifesting that Au@Cu_2-x_Se NPs and NIR could collaboratively enhance the ROS content in tumor cells. Next, mitochondrial hydroxyl radical detection assay kit was used to specifically detect the generation of ·OH radicals at cell level. The cell-permeable OH580 probe can selectively react with ·OH present in live cells to generate a red fluorescence signal. As shown in Fig. 3C, Au@Cu_2-x_Se + H_2_O_2_ + NIR group showed a significantly higher fluorescence signal than other groups, further confirming that photothermal can accelerate the Fenton-like reaction catalyzed by Au@Cu_2-x_Se NPs to produce abundant ·OH radicals in tumor cells.

**Fig. 4:**
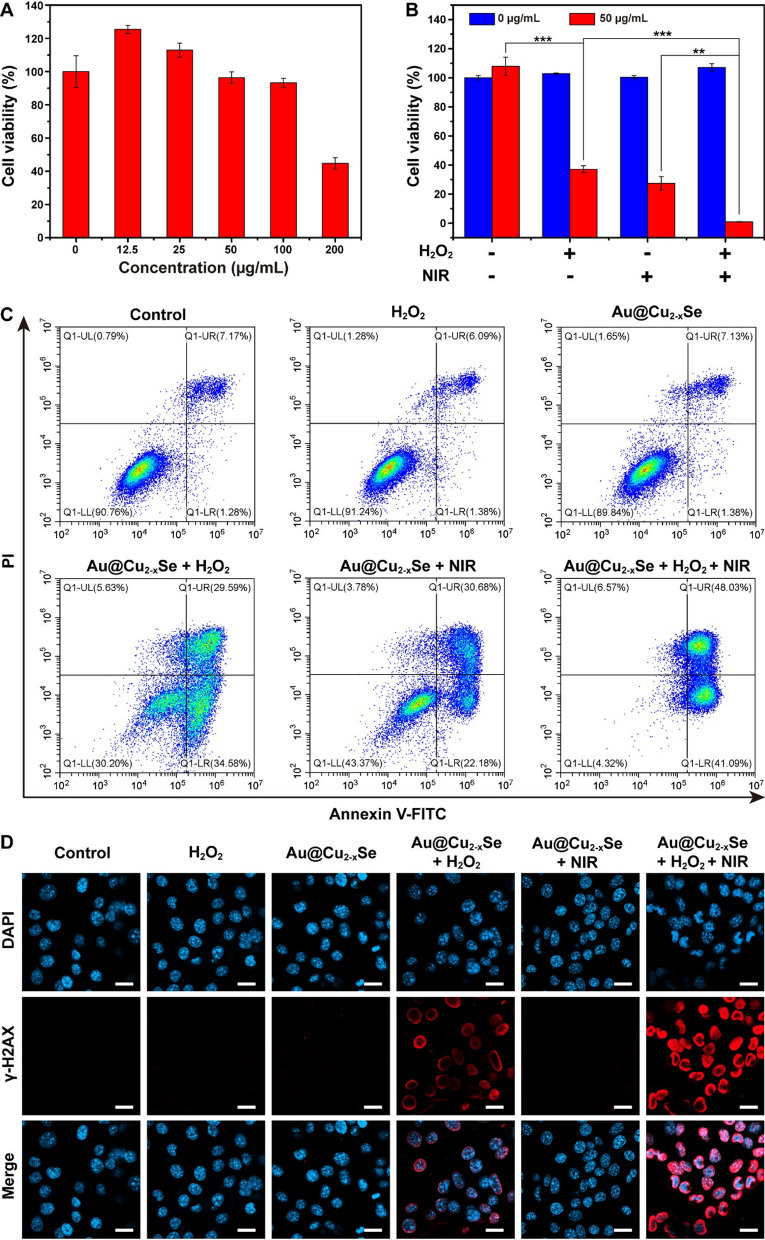
** A** Cytotoxicity of Au@Cu_2−x_Se NPs toward 4T1 tumor cells at different concentrations. **B** 4T1 tumor cell viability after different treatments. Data are presented as mean ± s.d. (n = 6). *P < 0.05, **P < 0.01, ***P < 0.001. **C** Flow cytometric analysis of 4T1 tumor cells after different treatments. **D** CLSM images of 4T1 tumor cells after different treatments, where DAPI (blue) and γ-H2AX (red) were used to observe the cell nucleus and DAN damage, respectively. Scale bar = 20 μm. Au@Cu_2−x_Se: 50 µg mL^−1^. H_2_O_2_: 100 µM. NIR: 808 nm laser, 1.0 W cm^−2^ for 5 min

The biocompatibility of nanoagent is another important criterion for biomedical applications. Cytotoxicity of Au@Cu_2-x_Se NPs on HEK293 normal cells and 4T1 tumor cells was then investigated. Various concentrations of Au@Cu_2-x_Se NPs were incubated with cells for 24 h. Then the cell viability was monitored by Cell Counting Kit-8 (CCK-8) assay. Negligible cytotoxicity to both normal cells (Additional file 1: Fig. S13) and tumor cells (Fig. 4A) were observed after incubation with Au@Cu_2-x_Se NPs at low concentration (≤ 100 µg mL^−1^). Interestingly, when the concentration of Au@Cu_2-x_Se NPs was below 50 µg mL^−1^, the cell viability of 4T1 tumor cells was higher than that of control group (0 µg mL^−1^). According to previous reports, ROS with a proper concentration is a messenger to mediate the normal physiological process, while excess ROS can destroy the antioxidant system of cell and induce cell death [1–3]. Noting that there are trace amounts of H_2_O_2_ in tumor cell, small amount of ·OH will be generated through Fenton-like reaction after introducing Au@Cu_2-x_Se with low concentration. So, the trace ·OH as a messenger might promote cell growth [1, 2]. When the concentration of Au@Cu_2-x_Se reached 200 µg mL^−1^, cell viability began to decline. These results suggest good biocompatibility of Au@Cu_2-x_Se at low concentration.

The cellular uptake ability of Au@Cu_2-x_Se NPs was also examined. After incubated with Au@Cu_2-x_Se NPs for various times, the content of Au in 4T1 tumor cells was quantified by inductively coupled plasma mass spectrometry (ICP-MS). Additional file 1: Fig. S14 presents that Au@Cu_2-x_Se NPs could be effectively internalized by tumor cells, and the amount of ingestion increased with increasing incubation time.

A relatively low concentration (50 µg mL^−1^) was chosen to investigate the in vitro anti-proliferation effect of Au@Cu_2-x_Se NPs via PTT + CDT (Fig. 4B). Incubation of 4T1 tumor cells with Au@Cu_2-x_Se, H_2_O_2_, or NIR alone showed negligible influence on cell viability. When cells were treated with either Au@Cu_2-x_Se + NIR or Au@Cu_2-x_Se + H_2_O_2_, substantial decrease in cell viability was observed. Especially, cells treated with Au@Cu_2-x_Se + H_2_O_2_ + NIR exhibited much more apparent decline in cell viability, showing the synergistically enhanced antitumor effect. CLSM was then employed to visualize the cell killing induced by hyperthermia and ·OH. Cells cultured under different conditions were co-stained with Calcein AM (living cells staining, green fluorescence) and PI (dying cells staining, red fluorescence). The CLSM images (Additional file 1: Fig. S15) revealed that a portion of 4T1 tumor cells was killed cultured under the condition of either Au@Cu_2-x_Se + H_2_O_2_ or Au@Cu_2-x_Se + NIR. Reasonably, when cells were treated with Au@Cu_2-x_Se + H_2_O_2_ + NIR, almost all cells were dead. Subsequently, an Annexin V-FITC/PI method was carried out by flow cytometry to characterize cell apoptosis and necrosis after different treatments. Figure 4C presents that cells treated with Au@Cu_2-x_Se + H_2_O_2_ + NIR showed the most obvious late apoptosis or necrosis than cells treated with other treatments. Moreover, immunofluorescent staining of γ-H2AX found that Au@Cu_2-x_Se + H_2_O_2_ treatment induced the formation of apoptotic ring (Fig. 4D). According to previous report, nuclear γ-H2AX apoptotic ring, which can be detected in early apoptotic cells, is usually caused by early DNA breaks at the nuclear periphery [26]. So we speculate that the ·OH generated outside the nucleus attacks DNA at the nuclear periphery and initiates cell apoptosis. Of course, Au@Cu_2-x_Se + H_2_O_2_ + NIR treatment caused more serious DNA damage.

### In vivo biosafety of Au@Cu_2-x_Se NPs

**Fig. 5:**
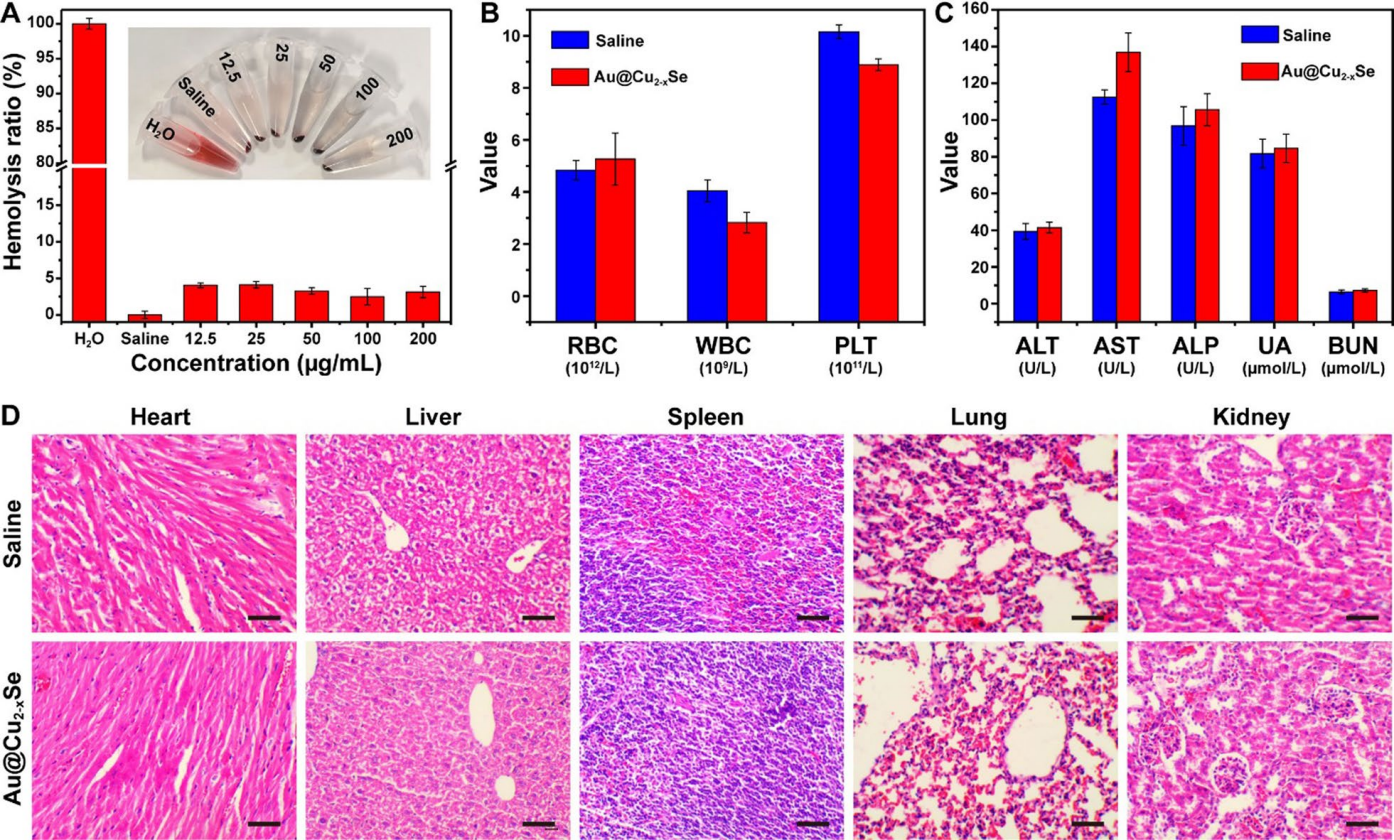
** A** Hemolysis ratio of red blood cells incubated with Au@Cu_2-x_Se NPs at various concentrations for 5 h. **B** Routing blood analysis and **C** hepatic and kidney function analysis of control and experiment groups for in vivo toxicity evaluation after intravenous injection of saline or Au@Cu_2-x_Se NPs. Data are presented as mean ± s.d. (n = 3). **D** H&E staining on major organ tissues of control and experiment groups for in vivo toxicity evaluation after intravenous injection of saline or Au@Cu_2-x_Se NPs. Scale bar = 50 μm. Saline: 200 µL. Au@Cu_2-x_Se: 2 mg mL^−1^ in saline, 200 µL

It is necessary to test the biosafety of Au@Cu_2-x_Se NPs before in vivo application. Hemolysis assay was firstly conducted to investigate the influence of Au@Cu_2-x_Se NPs on hemolysis of red blood cells, using pure water and saline as positive and negative controls, respectively. The calculated hemolysis ratios were less than 5% at all concentrations (Fig. 5A), indicating the acceptable blood compatibility of Au@Cu_2-x_Se NPs. After saline (control group) and Au@Cu_2-x_Se suspended in saline (2 mg mL^−1^, 200 µL) were injected into mice via intravenous injection, mice were observed for 14 days. The mice in Au@Cu_2-x_Se NPs group revealed no evident weight decrease, confirming the low systemic toxicity of Au@Cu_2-x_Se NPs (Additional file 1: Fig. S16). To determine the long-term toxicity of Au@Cu_2-x_Se NPs, hematological biochemistry indexes were analyzed at 14 d. The changes of routine blood parameters, including red blood cells (RBC), white blood cells (WBC), platelets (PLT), were within acceptable ranges (Fig. 5B). Hepatic and kidney function indexes, including alanine aminotransferase (ALT), aspartate aminotransferase (AST), alkaline phosphatase (ALP), uric acid (UA), and blood urea nitrogen (BUN), fell into normal range (Fig. 5C). Hematoxylin and eosin (H&E) staining analyses on major organ tissues (i.e., heart, liver, spleen, lung, and kidney) were also conducted. H&E results revealed no observable tissue damage or inflammatory lesions (Fig. 5D). Taken together, such experiments confirm that Au@Cu_2-x_Se NPs provided insignificant systemic toxicity under the treatment dose, strengthening their biomedical utilization as a safe theranostic nanoagent.

### In vitro and in vivo PA/CT imaging of Au@Cu_2-x_Se NPs

**Fig. 6:**
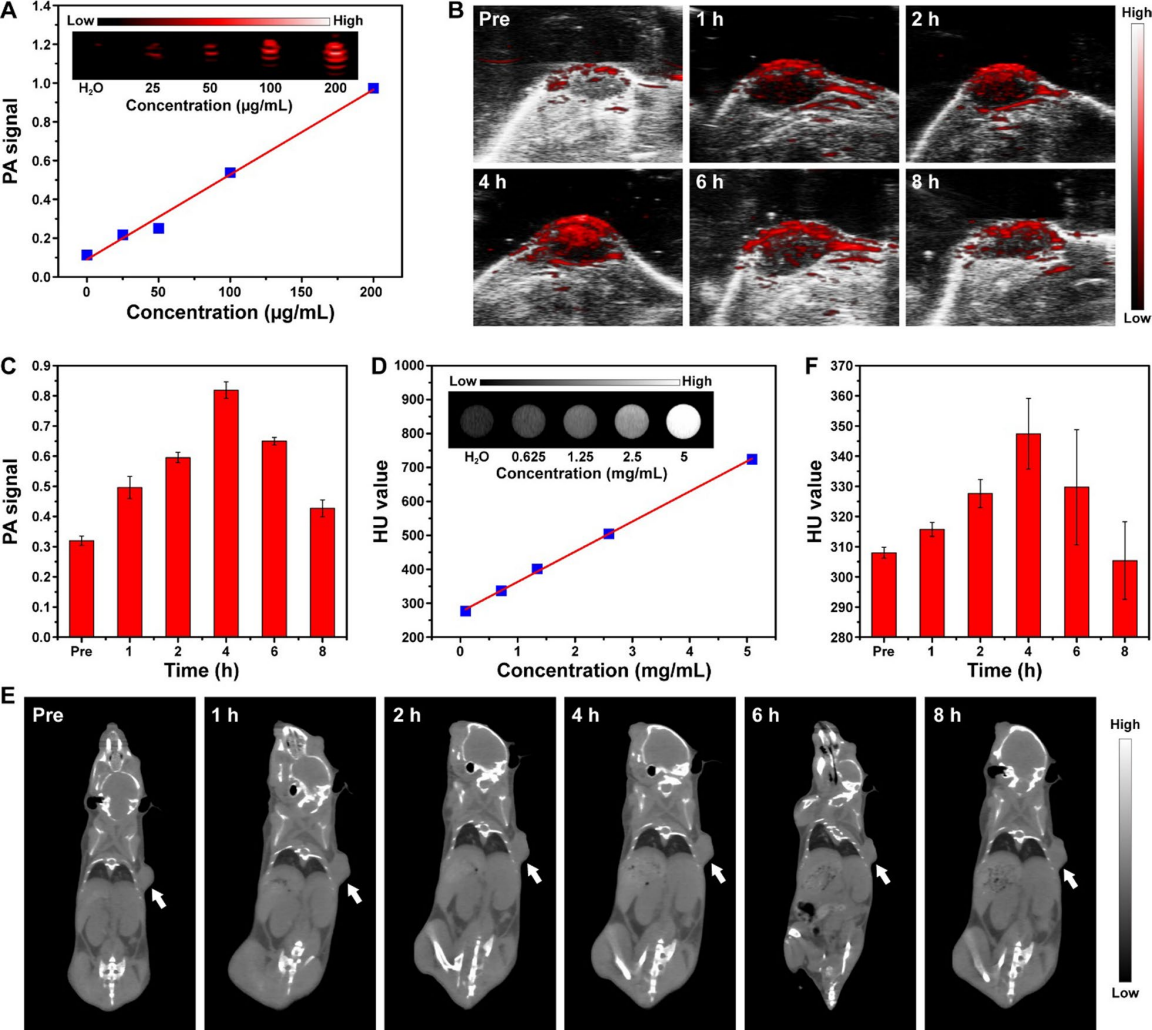
** A** In vitro PA images and corresponding signal intensities of Au@Cu_2-x_Se aqueous dispersions with different concentrations. **B** In vivo PA images of tumor and **C** corresponding signal intensities obtained at different time points after intravenous injection of Au@Cu_2-x_Se NPs (2 mg mL^−1^ in saline, 200 µL). **D** In vitro CT images and corresponding HU values of Au@Cu_2-x_Se aqueous dispersions with different concentrations. **E** In vivo CT images of tumor (indicated by white arrow) and **F** corresponding HU values obtained at different time points after intravenous injection of Au@Cu_2-x_Se NPs (2 mg mL^−1^ in saline, 200 µL). Data are presented as mean ± s.d. (n = 3)

The high photothermal conversion efficiency means that Au@Cu_2-x_Se NPs could be a contrast agent for PA imaging. The PA imaging performance of Au@Cu_2-x_Se NPs was then evaluated on an ultrasound-photoacoustic dual-mode imaging system, wherein the excitation wavelength for PA was 808 nm. Figure 6A presents that the in vitro PA signal increased linearly with the increase of concentration of Au@Cu_2-x_Se NPs. After saline containing Au@Cu_2-x_Se NPs (2 mg mL^−1^, 200 µL) was intravenously injected into 4T1 tumor-bearing mice, the PA signal (labeled with red) in the tumor region (localized by ultrasonic signal labeled with gray) was gradually enhanced to the maximum at around 4 h post-injection (Fig. 6B, C), which might be ascribed to the enhanced permeability and retention (EPR) effect of NPs in tumor. The maximal PA signal was 2.6 times higher than that acquired before the injection of Au@Cu_2-x_Se NPs (Fig. 6 C). The time-dependent PA image indicates that Au@Cu_2-x_Se NPs reached the maximum accumulation in tumor at 4 h after intravenous injection.

Au is a high-Z element and has the capability of X-ray attenuation. So the CT imaging performance of Au@Cu_2-x_Se NPs was also assessed. The imaging brightness and Hounsfield unit (HU) values of Au@Cu_2-x_Se aqueous dispersions increased with the increasing NPs concentration (Fig. 6D). After saline containing Au@Cu_2-x_Se NPs (2 mg mL^−1^, 200 µL) was administrated into tumor-bearing mice intravenously, the HU value at tumor site increased from 308.0 before injection to 347.4 after 4 h injection (Fig. 6E, F). When changing the injection method from intravenous injection to intratumoral injection, a strong whitening effect was observed (Additional file 1: Fig. S17). These results make us believe that Au@Cu_2-x_Se NPs have the potential to be a contrast-enhancing agent for CT imaging.

### In vivo photothermal and chemodynamic therapy based on Au@Cu_2-x_Se NPs

**Fig. 7:**
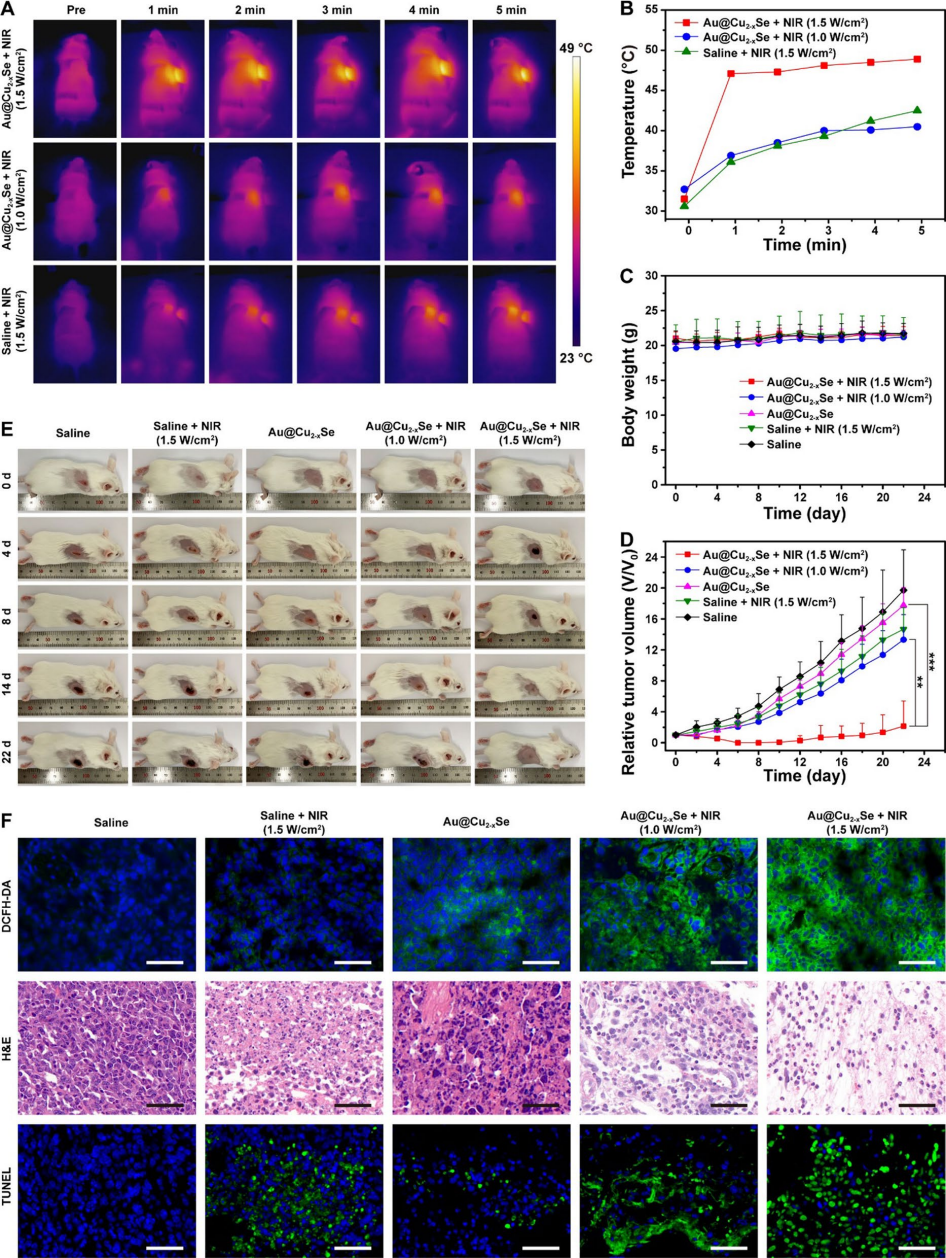
** A** Thermal images and **B** temperature rise curves at tumor sites of 4T1 tumor-bearing mice after intravenous injection of saline or Au@Cu_2-x_Se NPs followed by NIR irradiation. **C** Body weight changes, **D** tumor volume growth curves, and **E** digital photographs of 4T1 tumor-bearing mice after intravenous injection of saline or Au@Cu_2-x_Se NPs followed by NIR irradiation. Data are presented as mean ± s.d. (n = 5). *P < 0.05, **P < 0.01, ***P < 0.001. **F** DCFH-DA, H&E, and TUNEL staining of tumor tissues after corresponding treatments, where blue represents the cell nucleus stained with DAPI. Scale bar = 50 μm. Saline: 200 µL. Au@Cu_2-x_Se: 2 mg mL^−1^ in saline, 200 µL. NIR: 808 nm laser, 1.0 or 1.5 W cm^−2^ for 5 min

Inspired by the in vitro anticancer effects, the in vivo therapeutic effect of PTT + CDT based on Au@Cu_2-x_Se NPs was examined by establishing 4T1 tumor-bearing mice. Tumor-bearing mice were divided randomly into five groups: (1) saline; (2) saline + NIR (1.5 W cm^−2^); (3) Au@Cu_2-x_Se; (4) Au@Cu_2-x_Se + NIR (1.0 W cm^−2^); (5) Au@Cu_2-x_Se + NIR (1.5 W cm^−2^). The NIR irradiation was administrated at 4 h after intravenous injection of Au@Cu_2-x_Se NPs suspended in saline, since PA and CT imaging showed maximal accumulation of Au@Cu_2-x_Se NPs in tumor at this time point. The tumor temperature in Au@Cu_2-x_Se + NIR (1.5 W cm^−2^) group rapidly increased from 31.5 to 48.9 °C in 5 min of 808 nm NIR irradiation at the power density of 1.5 W cm^−2^, which was higher than that in saline + NIR (1.5 W cm^−2^) group under the same NIR power density (Fig. 7A, B). These validate the effective intratumoral accumulation of Au@Cu_2-x_Se NPs and their excellent in vivo photothermal conversion performance. During the therapeutic period, all mice in each group displayed no apparent decrease in body weight (Fig. 7C), H&E staining on major organs from all groups showed no obvious damages (Additional file 1: Fig. S18), implying the negligible adverse effects of these treatments on mice. As for the suppressing performance toward tumor growth, Au@Cu_2-x_Se + NIR (1.5 W cm^−2^) treatment was much more effective than other treatments (Fig. 7D, E and Additional file 1: Fig. S19), ascribing to the synergistic enhancement of PTT and boosted CDT.

To confirm the in vivo photothermal enhanced CDT effect, tumors in saline + NIR (1.5 W cm^−2^) group were irradiated with NIR at 1.5 W cm^−2^, while tumors in Au@Cu_2-x_Se + NIR (1.0 W cm^−2^) group were irradiated with NIR at 1.0 W cm^−2^. The tumor temperature in Au@Cu_2-x_Se + NIR (1.0 W cm^−2^) group increased from 32.7 to 40.5 °C in 5 min of NIR irradiation (Fig. 7A, B). A similar tumor temperature increase was also observed in saline + NIR (1.5 W cm^−2^) group (Fig. 7A, B). So these treatments could ensure that the temperature conditions at tumor sites in these two groups were close to each other. The inhibition efficiency of tumor growth in Au@Cu_2-x_Se + NIR (1.0 W cm^−2^) group was higher than that of both Au@Cu_2-x_Se group and saline + NIR (1.5 W cm^−2^) group (Fig. 7D, E). Considering that the tumor temperature in Au@Cu_2-x_Se + NIR (1.0 W cm^−2^) group was higher than that in Au@Cu_2-x_Se group and was close to that in saline + NIR (1.5 W cm^−2^) group, the anti-tumor effect of Au@Cu_2-x_Se + NIR (1.0 W cm^−2^) could be attributed to the photothermal enhanced CDT effect. However, tumors in Au@Cu_2-x_Se + NIR (1.0 W cm^−2^) group were inhibited, but were not eliminated, suggesting that the insufficient ·OH and mild photothermal produced in this group were not able to completely destroy the tumor. When the NIR power density was increased to 1.5 W cm^−2^, that was, Au@Cu_2-x_Se + NIR (1.5 W cm^−2^) group, the tumors could be completely suppressed in the first eight days, substantiating the synergistic enhancement of PTT and boosted CDT in vivo.

The tumors from the representative mouse in each group after NIR irradiation were collected to verify the generation of ROS by using DCFH-DA as ROS probe. As presented in Fig. 7F, unlike saline group and saline + NIR group, both of which showed undetectable green fluorescence corresponding to ROS, Au@Cu_2-x_Se group exhibits a weak green fluorescence, while Au@Cu_2-x_Se + NIR (1.0 W cm^−2^) group and Au@Cu_2-x_Se + NIR (1.5 W cm^−2^) group displayed an obvious enhanced green fluorescence. These results may be attributed to the fact that the intratumorally accumulated Au@Cu_2-x_Se NPs could induce the generation of ROS in tumor through photothermal-assisted Fenton-like reaction [27].

It should be noted that the tumor growth in Au@Cu_2-x_Se group was not suppressed after single CDT treatment (Fig. 7D). The reason for this phenomenon may be that the in vivo Fenton-like kinetics catalyzed by Au@Cu_2-x_Se only are not fast enough without the assistance of exogenous energy, so that the cell damage caused by in situ generated ·OH could be repaired by the oxidative damage repair system in the first few days after CDT treatment. To prove this supposition, H&E staining, as well as terminal deoxynucleotidyl transferase-mediated dUTP nick-end labeling (TUNEL) staining of tumor tissues, were performed to detect cell damage after different therapies. To avoid the complete repair of oxidative damage, the tumor from the representative mouse in each group at 20 h post-treatment was collected. As shown in Fig. 7 F, a slight portion of necrosis and apoptosis was observed in Au@Cu_2-x_Se group compared with that in saline group, implying that single CDT treatment might cause cell damage in the early stage. The extent and severity of necrosis and apoptosis in Au@Cu_2-x_Se + NIR (1.0 W cm^−2^) group were higher than those in both Au@Cu_2-x_Se group and saline + NIR (1.5 W cm^−2^) group, validating the photothermal enhanced CDT effect. Of course, the Au@Cu_2-x_Se NPs + NIR (1.5 W cm^−2^) group had the most severe cell death.

Intratumoral injection of Au@Cu_2-x_Se NPs was also conducted to evaluate the in vivo synergistic therapy of PTT + CDT. The tumor temperature in Au@Cu_2-x_Se + NIR group rapidly increased from 33.1 to 57.7 °C in 5 min of 808 nm NIR irradiation at 0.5 W cm^−2^, while no obvious temperature increase was observed in saline + NIR group under the same NIR condition (Additional file 1: Fig. S20). During the therapeutic period, all mice in each group displayed no apparent decrease in body weight (Additional file 1: Fig. S21A). As for the suppressing performance toward tumor growth, Au@Cu_2-x_Se + NIR treatment was much more effective than other treatments, and the tumors in this group were completely eradicated after 16 days (Additional file 1: Fig. S21B, C). H&E and TUNEL staining on tumors collected at 20 h post-treatment revealed considerably increased necrosis and apoptosis of tumor cells in Au@Cu_2-x_Se + NIR group (Additional file 1: Fig. S22).

## Conclusions

In summary, we have exploited the application of core-shell Au@Cu_2-x_Se NPs acting as a theranostic nanoagent for PA/CT imaging-mediating synergistic therapy of PTT + CDT in the field of cancer treatment. The Au@Cu_2-x_Se NPs were fabricated through a facile two-step reduction method. Biosafety study certificated the qualification of Au@Cu_2-x_Se NPs in biocompatibility. Imaging assessment indicated that Au@Cu_2-x_Se NPs could be a contrast agent for PA and CT imaging. In vitro and in vivo examinations revealed that the high photothermal conversion efficiency of Au@Cu_2-x_Se NPs not only permits the PTT efficacy but also facilitates the CDT effect assisted by the heat generated in PTT process, thus clarifying a combinational therapeutic PTT + CDT outcome for the inhibition of tumor growth. This versatile agent may provide a paradigm to realize an accurate and noninvasive theranostic approach for cancer treatment.

## Experimental section

### Materials

Hydrogen Tetrachloroaurate(III) Trihydrate (HAuCl_4_·3H_2_O) and methylene blue (MB) were obtained from Shanghai Titan Technology Co., Ltd. Copper(II) sulfate pentahydrate (CuSO_4_·5H_2_O), selenium dioxide (SeO_2_), sodium citrate dihydrate, ascorbic acid, and hydrogen peroxide (H_2_O_2_) were purchased from Sinopharm Chemical Reagent Co., Ltd. Polyvinylpyrrolidone (PVP, Mw ~55,000), 2′,7′- dichlorofluorescein diacetate (DCFH-DA), and Calcein AM were provided by Sigma Aldrich. Roswell Park Memorial Institute 1640 (RPMI-1640) was supported by Corning Incorporated. Fetal bovine serum (FBS), trypsin, and penicillin-streptomycin solution were supplied from Gibco Company. Cell Counting Kit-8 (CCK-8) solution was purchased from Dojindo Laboratories. Hoechst 33,342, 4′,6-diamidino-2-phenylindole (DAPI), and propidium iodide (PI) were acquired from Beyotime Biotechnology. Mitochondrial hydroxyl radical detection assay, anti-gamma H2AX (phospho S139) antibody [EP854(2)Y], and goat polyclonal secondary antibody to rabbit IgG - H&L (Alexa Fluor® 647) were purchased from Abcam Company. Accutase™ Cell Detachment Solution and FITC Annexin V Apoptosis Detection Kit were supplied from BD Bioscience. All chemicals and reagents were used as received without further purification.

### Synthesis of Au NPs

HAuCl_4_ aqueous solution prepared by mixing 1.215 mL of 10 mM HAuCl_4_ solution and 50 mL of deionized water was heated to slight boiling. A condenser was utilized to prevent the evaporation of the water. Then 50 mL of 10 mg mL^−1^ sodium citrate solution was added into the boiling HAuCl_4_ solution. After keeping boiling for 15 min under continuous stirring, the mixture was allowed to cool to room temperature. The resulted Au NPs were collected by centrifuging the suspension at 15,000 rpm for 15 min.

### Synthesis of Au@Cu_2-x_Se NPs

5.5 mL of Au NPs redispersed in deionized water was mixed with 1.6 mL of 10 mg mL^−1^ PVP solution and kept stirring at 30 °C for 1 h, followed by the addition of 0.1 mL of 0.1 M SeO_2_ and 0.3 mL of 0.2 M ascorbic acid solution. After reaction for 30 min, a mixed solution of 0.1 mL of 0.2 M CuSO_4_ and 0.4 mL of 0.2 M ascorbic acid was added. After stirring the suspension at 30 °C for another 10 h, the Au@Cu_2-x_Se NPs were collected by centrifuging the suspension at 15,000 rpm for 15 min and washed three times with deionized water.

### Synthesis of Cu_2-x_Se NPs

1.6 mL of 10 mg mL^−1^ PVP solution was added into 5.5 mL of deionized water and kept stirring at 30 °C, followed by the addition of 0.1 mL of 0.1 M SeO_2_ and 0.3 mL of 0.2 M ascorbic acid solution. After reaction for 30 min, a mixed solution of 0.1 mL of 0.2 M CuSO_4_ and 0.4 mL of 0.2 M ascorbic acid was added. After stirring the suspension at 30 °C for another 10 h, the Cu_2-x_Se NPs were collected by centrifuging the suspension at 15,000 rpm for 15 min and washed three times with deionized water.

### Characterization

The morphology and structure were characterized by transmission electron microscopy (TEM, JEOL JEM-2100 F). The crystal structure was determined by X-ray diffraction (XRD, Rigaku D/max2500). The chemical state was analyzed by X-ray photoelectron spectroscopy (XPS, Thermo Escalab 250), and the binding energy of C 1 s peak at 284.8 eV was taken as an internal standard. Fourier transform infrared (FTIR) spectrum was recorded on Bruker Tensor-27 spectrometer. Ultraviolet-visible-infrared (UV-vis-NIR) absorption spectrum was scanned on Persee TU-1901 spectrometer. The metal element content was determined by inductively coupled plasma optical emission spectroscopy (ICP-OES, Varian 710ES). Hydrodynamic size and zeta potential were characterized by a particle size zeta potential analyzer (Malvern ZEN3690).

### Photothermal performance

An 808 NIR laser was used to estimate the photothermal performance of Au@Cu_2-x_Se NPs. The NIR power density was maintained at 1.0 W cm^−2^ in all tests. The volume of Au@Cu_2-x_Se aqueous dispersions in each test was 1.5 mL. Temperature changes were recorded by a thermal camera. For concentration-dependent photothermal performance, various Au@Cu_2-x_Se aqueous dispersions in a quartz cuvette with concentrations of 0, 25, 50, 100, and 200 µg mL^−1^ were exposed to laser for 10 min. To evaluate the photothermal stability, Au@Cu_2-x_Se aqueous dispersion with a concentration of 50 µg mL^−1^ was irradiated by laser for 10 min (laser on) followed by natural cooling without irradiation for another 10 min (laser off). Such heating/cooling processes were repeated 6 times. Heating-cooling curve was obtained by continuously irradiating 50 µg mL^−1^ dispersion with laser for 15 min, and then turning off the laser to allow the dispersion to naturally cool to room temperature. Following previously reported method [8, 20, 28], the photothermal conversion efficiency was calculated according to Eqs. 1–4.

1 $$\eta =\frac{{hS({T_{\hbox{max} }} - {T_{\hbox{max} ,{{\text{H}}_{\text{2}}}{\text{O}}}})}}{{I(1 - {{10}^{ - {A_\lambda }}})}}$$

2 $$hS=\frac{{m{C_{\text{p}}}}}{{{\tau _s}}}$$

3 $$t= - {\tau _{\text{s}}}\ln \theta$$

4 $$\theta =\frac{{T - {T_{{\text{sur}}}}}}{{{T_{\hbox{max} }} - {T_{{\text{sur}}}}}}$$

where *η* is the photothermal conversion efficiency, *h* is the heat-transfer coefficient, *S* is the surface area of container, *T* is the temperature of Au@Cu_2-x_Se aqueous dispersion, *T*_max_ is the maximum temperature of Au@Cu_2-x_Se aqueous dispersion, *T*_max,H2O_ is the maximum temperature of pure water, *T*_sur_ is the ambient temperature of surroundings, *I* is the laser power, *A*_λ_ is the absorbance of Au@Cu_2-x_Se aqueous dispersion at the wavelength of 808 nm, *m* and *C*_p_ are the mass (1.5 g) and heat capacity (4.2 J g^−1^) of solvent (water), *t* is the cooling time, *τ*_s_ is the time constant of sample system, *θ* is the driving force temperature. *τ*_s_ can be obtained by applying the linear time data from the cooling period vs. –ln*θ* (Fig. 2D, E).

### Photothermal enhanced Fenton-like catalytic performance

An MB degradation assay was used to monitor the ·OH generation. First, mixed aqueous solutions of Au@Cu_2-x_Se and MB remained static state in the dark for 30 min at 25, 37, and 42 °C, respectively. Second, after adding H_2_O_2_, the mixtures were then remained static state in the dark for another 30 min at 25, 37, or 42 °C, respectively. The final concentrations of Au@Cu_2-x_Se NPs, MB, and H_2_O_2_ in mixtures were set at 100 µg mL^−1^, 10 µg mL^−1^, and 10 mM, respectively. Finally, after removing Au@Cu_2-x_Se NPs via centrifugation at 15,000 rpm for 15 min, the absorbance of each supernatant at 664 nm was measured. In these experiments, the pure MB solution (10 µg mL^−1^), and the mixture of MB (10 µg mL^−1^) and H_2_O_2_ (10 mM) solution, were chosen as control.

Next, ESR was used to confirm the generation of ·OH in Fenton-like reaction. DMPO was used as a spin trap agent for ·OH. After keeping the mixed aqueous solutions of Au@Cu_2-x_Se, H_2_O_2_, and DMPO for 5 min at 25, 37, and 42 °C, the mixtures were used to record ESR spectra by a spectrometer (Bruker ELEXSYS E500, CW X-band), respectively. The final concentrations of Au@Cu_2-x_Se, H_2_O_2_, and DMPO were set at 50 µg mL^−1^, 100 µM, and 50 mM, respectively. The mixture of Au@Cu_2-x_Se (50 µg mL^−1^) and DMPO (50 mM), and the mixture of H_2_O_2_ (100 µM) and DMPO (50 mM), were chosen as control.

To prove the photothermal accelerated ·OH generation in Fenton-like reaction, 1.5 mL of mixed aqueous solution of Au@Cu_2-x_Se NPs, MB, and H_2_O_2_ was irradiated with or without 808 nm NIR laser at 1.0 W cm^−2^ for 10 min. The final concentrations of Au@Cu_2-x_Se NPs, MB, and H_2_O_2_ in mixture were set at 100 µg mL^−1^, 10 µg mL^−1^, and 10 mM, respectively. The Temperature rise was recorded by thermal camera. Finally, after removing Au@Cu_2-x_Se NPs via centrifugation at 15,000 rpm for 15 min, the absorbance of supernatant at 664 nm was measured.

### Detection of intracellular ROS

CLSM (Zesis LSM800) was adopted to detect the intracellular ROS by using DCFH-DA (Sigma-Aldrich) as ROS probe. Cells were divided into six groups, including (1) control, (2) H_2_O_2_, (3) Au@Cu_2-x_Se, (4) Au@Cu_2-x_Se + H_2_O_2_, (5) Au@Cu_2-x_Se + NIR, and (6) Au@Cu_2-x_Se + H_2_O_2_ + NIR. First, 2 × 10^5^ 4T1 tumor cells were seeded into the CLSM-specific culture disk and then cultured at 37 °C under 5% CO_2_ for 24 h. Second, after replacing the culture medium with 1 mL of FBS-free RPMI-1640 medium containing Au@Cu_2-x_Se (50 µg mL^−1^) or H_2_O_2_ (100 µM), the cells in group (5) and (6) were irradiated with NIR laser (1.0 W cm^−2^) for 5 min, respectively. After irradiation, the cells continued to incubate for 30 min. Third, after washing the cells three times with PBS, the cells were incubated with 1 mL of FBS-free RPMI-1640 medium containing DCFH-DA (10 µM) and Hoechst 33,342 (5 µg mL^−1^) for 20 min. Fourth, the cells were washed three times with PBS followed by the addition of 1 mL of FBS-free RPMI-1640 medium. Then the fluorescence images of cells were collected by CLSM.

### Detection of intracellular ·OH radicals

Mitochondrial hydroxyl radical detection assay kit (Abcam) was used to detect the intracellular ·OH. First, 2 × 10^5^ 4T1 tumor cells were seeded into the CLSM-specific culture disk and then cultured for 24 h. Second, after replacing the culture medium with 200 µL of the prepared OH580 stain working solution, the cells continued to incubate for 1 h without light. Third, after washing the cells one time with PBS, the cells were incubated with 1 mL of RPMI-1640 complete medium containing Au@Cu_2-x_Se (50 µg mL^−1^) or H_2_O_2_ (100 µM), and then irradiated with or without NIR laser (1.0 W cm^−2^) for 5 min. After irradiation, the cells continued to incubate for 30 min. Fourth, the cells were incubated with 1 mL of PBS containing Hoechst 33,342 (5 µg mL^−1^) for 10 min. Fifth, the cells were washed three times with PBS followed by the addition of 1 mL assay buffer. Then the fluorescence images of cells were collected by CLSM.

### In vitro cytotoxicity assay

The cytotoxicity of Au@Cu_2-x_Se against HEK293 normal cells and 4T1 tumor cells were examined via a CCK-8 assay. HEK293 normal cells or 4T1 tumor cells were seeded into 96-well plate at 1 × 10^4^ cells/well and incubated for 24 h. Then the cells were incubated in 100 µL of the complete medium containing Au@Cu_2-x_Se NPs at various concentrations of 0, 12.5, 25, 50, 100, and 200 µg mL^−1^. After 24 h, the culture medium was removed and the cells were washed three times with PBS. Then the cells were incubated in 100 µL of complete medium containing 10 µL CCK-8. After another 1–2 h, the optical densities of each well were recorded at 450 nm on a microplate reader (BioTek Cytation 3).

### In vitro cellular uptake

First, 4T1 tumor cells were seeded into 6-well plate at 1.5 × 10^5^ cells/well and then cultured at 37 °C under 5% CO_2_ for 24 h. Second, after replacing the culture medium with 1 mL of the complete medium containing Au@Cu_2-x_Se NPs (50 µg mL^−1^), the cells were then incubated for 0.5, 1, 2, 4, and 8 h, respectively (three wells at each time point). Third, the culture medium was removed and the cells were washed three times with PBS, digested with 0.25% trypsin (Gibco), and centrifuged at 1500 rpm for 3 min. When the collected cells were redispersed in 1 mL PBS, the cells were counted. Finally, the content of Au in PBS was analyzed by ICP-MS.

### In vitro PTT and CDT

4T1 tumor cells were divided into eight groups, including (1) control, (2) H_2_O_2_, (3) NIR, (4) H_2_O_2_ + NIR, (5) Au@Cu_2-x_Se, (6) Au@Cu_2-x_Se + H_2_O_2_, (7) Au@Cu_2-x_Se + NIR, and (8) Au@Cu_2-x_Se + H_2_O_2_ + NIR. First, 4T1 tumor cells were seeded into 96-well plate at 1 × 10^4^ cells/well and incubated for 24 h. Second, after replacing the culture medium with 100 µL of the complete medium containing Au@Cu_2-x_Se (50 µg mL^−1^) or H_2_O_2_ (100 µM), the cells were irradiated with or without NIR laser (1.0 W cm^−2^) for 5 min. After irradiation, the cells continued to incubate for 24 h. Third, the culture medium was removed and the cells were washed three times with PBS. Then the cells were incubated in 100 µL of complete medium containing 10 µL CCK-8 for another 1 h. Finally, the optical densities of each well were recorded at 450 nm on a microplate reader.

CLSM was then employed to observe the cell death induced by hyperthermia and ·OH radicals. First, 2 × 10^5^ 4T1 tumor cells were seeded into the CLSM-specific culture disk and then cultured at 37 °C under 5% CO_2_ for 24 h. Second, after replacing the culture medium with 1 mL of the complete medium containing Au@Cu_2-x_Se (50 µg mL^−1^) or H_2_O_2_ (100 µM), the cells were irradiated with or without NIR laser (1.0 W cm^−2^) for 5 min. After irradiation, the cells continued to incubate for 4 h. Third, the culture medium was replaced by 1 mL of PBS containing Calcein AM (2 µM) and PI (4 µM) and co-incubated with the cells for another 20 min. Finally, the fluorescence images of cells were collected directly by CLSM.

### In vitro apoptosis and necrosis analysis by flow cytometry

First, 4T1 tumor cells were seeded into 12-well plate at 7 × 10^4^ cells/well and then cultured at 37 °C under 5% CO_2_ for 24 h. Second, after replacing the culture medium with 1 mL of the complete medium containing Au@Cu_2-x_Se (50 µg mL^−1^) or H_2_O_2_ (100 µM), the cells were irradiated with or without NIR laser (1.0 W cm^−2^) for 5 min. After irradiation, the cells continued to incubate for 24 h. Third, the cells were digested with Accutase™ Cell Detachment Solution (BD Biosciences) and re-suspended in PBS. Then, the cells were stained according to the FITC Annexin V Apoptosis Detection Kit (BD Biosciences) and quantified by flow cytometry (Beckman Cytoflex).

### In vitro DNA damage analysis

First, 2 × 10^5^ 4T1 tumor cells were seeded into the CLSM-specific culture disk and then cultured for 24 h. Second, after replacing the culture medium with 1 mL of RPMI-1640 complete medium containing Au@Cu_2-x_Se (50 µg mL^−1^) or H_2_O_2_ (100 µM), the cells were irradiated with or without NIR laser (1.0 W cm^−2^) for 5 min. After irradiation, the cells continued to incubate for 6 h. Third, these cells were fixed with 4% paraformaldehyde for 10 min, washed several times with PBS, permeabilized with immunostaining permeabilization buffer with triton X-100 (Beyotime Biotechnology) for 10 min, and blocked with QuickBlock blocking buffer (Beyotime Biotechnology) for 10 min at room temperature. Forth, after washing three times with PBS, the cells were stained with anti-gamma H2AX (phospho S139) antibody [EP854(2)Y] (Abcam, dilution 1:400) at 4 °C overnight. Fifth, after washing three times with PBS, the cells were incubated with the goat polyclonal secondary antibody to rabbit IgG-H&L (Alexa Fluor® 647) (Abcam, dilution 1:400) for 1 h. Sixth, after washing three times with PBS, the cells were stained with DAPI (Beyotime Biotechnology) for 5 min. Finally, the cells were washed three times with PBS followed by the addition of 1 mL PBS. Then the fluorescence images of cells were collected by CLSM.

### In vitro hemolysis assay

1 mL of fresh blood was taken from BALB/c mouse and stored in a tube containing EDTA. 2 mL of saline was added and mixed. Red blood cells (RBC) were collected by centrifuging the blood at 5000 rpm for 3 min. Then the RBC was washed three times with saline until the supernatant became colorless. After discarding the supernatant, 100 µL of RBC was taken and redispersed into 5 mL saline to prepare 2% volume fraction RBC suspension. Then a series of mixtures were prepared by mixing 500 µL RBC suspension and 500 µL saline containing Au@Cu_2-x_Se with different concentrations. The final concentrations of Au@Cu_2-x_Se in the mixtures were set at 12.5, 25, 50, 100, and 200 µg mL^−1^, respectively. The mixture of 500 µL RBC suspension and 500 µL saline, and the mixture of 500 µL RBC suspension and 500 µL pure water, were used as negative and positive controls, respectively. After remaining stable at room temperature for 5 h, these mixtures were centrifuged at 15,000 rpm for 15 min. Finally, the absorbance (A) of supernatant in different mixtures was recorded at 570 nm on microplate reader for calculating the hemolysis rate of RBC. Hemolysis rate was calculated according to Eq. 5.

5 $${\text{Hemolysis rate}}=\frac{{{{\text{A}}_{{\text{sample}}}} - {{\text{A}}_{{\text{saline}}}}}}{{{{\text{A}}_{{{\text{H}}_{\text{2}}}{\text{O}}}}{\text{-}}{{\text{A}}_{{\text{saline}}}}}} \times 100\%$$

### In vivo toxicity

After 200 µL of saline (control group) or 200 µL of Au@Cu_2-x_Se suspended in saline (2 mg mL^−1^) were injected into mice via intravenous injection (n = 3, per group), mice were sacrificed at 14 d to collect blood for hematological analyses and tissues (including heart, liver, spleen, lung, and kidney) for H&E stain. In these 14 days, weights of mice were measured every 2 days.

### Tumor model

100 µL PBS containing 4T1.2 cells (2 × 10^6^ cells mL^−1^) was subcutaneously injected beside the foreleg of a BALB/c female mouse (6-weeks old). When tumor grows to ~80 cm^3^, the tumor-bearing mouse was used in subsequent in vivo experiments.

### In vitro and in vivo PA imaging

PA imaging was performed on an ultrasound-photoacoustic dual-mode imaging system (VEVO LAZR-X, Fujifilm VisualSonics). The excitation wavelength for PA imaging was 808 nm. For in vitro PA imaging, various Au@Cu_2-x_Se aqueous dispersions with concentrations of 0, 25, 50, 100, and 200 µg mL^−1^ were injected into polyethylene tubes for PA testing, respectively. For in vivo PA imaging, 200 µL saline containing Au@Cu_2-x_Se NPs (2 mg mL^−1^) were intravenously injected into tumor-bearing mice (n = 3). Then the ultrasound and photoacoustic signals at tumor site were recorded at 1, 2, 4, 6, and 8 h post-injection, respectively. The signals before injection were used as control.

### In vitro and in vivo CT imaging

CT imaging was collected from the Siemens Inveon PET/CT imaging system. For in vitro CT imaging, various Au@Cu_2-x_Se aqueous dispersions with concentrations of 0, 0.625, 1.25, 2.5, and 5 mg mL^−1^ were placed in small tubes for CT imaging. In vivo CT imaging was conducted with tumor-bearing mice (n = 3, per group) after intravenous or intratumoral injection of Au@Cu_2-x_Se NPs. For intravenous injection, the CT images were collected at 1, 2, 4, 6, and 8 h post-injection of 200 µL saline containing Au@Cu_2-x_Se NPs (2 mg mL^−1^). The CT images before injection were used as control. For intratumoral injection, the CT images were collected after injection of 150 µL saline containing Au@Cu_2-x_Se NPs with 0, 6, and 8 mg mL^−1^, respectively.

### In vivo PTT and CDT

Tumor-bearing mice were divided randomly into five groups (n = 5, per group): (1) saline; (2) saline + NIR (1.5 W cm^−2^); (3) Au@Cu_2-x_Se; (4) Au@Cu_2-x_Se + NIR (1.0 W cm^−2^); (5) Au@Cu_2-x_Se + NIR (1.5 W cm^−2^). Mice in group (1) and (2) were intravenously injected with 200 µL pure saline, while mice in group (3), (4), and (5) were intravenously injected with 200 µL saline containing Au@Cu_2-x_Se NPs (2 mg mL^−1^). At 4 h post-injection, tumors in group (2) and (5) were irradiated with 808 nm NIR laser for 5 min at 1.5 W cm^−2^, while tumors in group (4) were irradiated with 808 nm NIR laser for 5 min at 1.0 W cm^−2^. The temperature changes at tumor sites were recorded with a thermal camera. The length and width of tumors and weight of mice were measured every 2 days. The tumor volume (V) was calculated according to equation V = (width^2^ × length)/2. The relative tumor volume (V/V_0_) was obtained by normalizing the tumor volume to the initial tumor volume (V_0_). Besides, the tumors at 20 h post-treatment were collected for H&E and TUNEL analyses. For in vivo ROS detection in tumor, 200 µL saline containing DCFH-DA (100 µM) was intratumorally injected into the tumor-bearing mice at 3.5 h post-injection of saline or Au@Cu_2-x_Se NPs. 30 min later, NIR irradiation was administrated. Then, tumors in each group were collected for fluorescence analysis.

Intratumoral injection of Au@Cu_2-x_Se NPs was also carried out to evaluate synergistic therapy of PTT + CDT. Tumor-bearing mice were divided randomly into four groups (n = 5, per group): (1) saline; (2) saline + NIR; (3) Au@Cu_2-x_Se; (4) Au@Cu_2-x_Se + NIR. After intratumoral injection of 100 µL pure saline or 100 µL saline containing Au@Cu_2-x_Se NPs (2 mg mL^−1^), tumors in group (2) and (4) were then irradiated with 808 nm NIR laser for 5 min at 0.5 W cm^−2^. Tumors at 20 h post-treatment were collected for H&E and TUNEL analyses.

### Statistical analysis

Bars display mean ± s.d., and statistical analysis was performed using Student’s t-test and the P values were provided (***P < 0.001; **P < 0.01; *P < 0.05).

## Supplementary Information

**Additional file 1.** Additional tables and figures.

## Data Availability

All data generated or analyzed during this study are included in this published article and its Additional files.
