# Supplementary material for: A core-shell Au@Cu2-xSe heterogeneous metal nanocomposite for photoacoustic and computed tomography dual-imaging-guided photothermal boosted chemodynamic therapy
Source: J Nanobiotechnology. 2021 Dec 7;19:410. doi: 10.1186/s12951-021-01159-x (PMC8650506; doi:10.1186/s12951-021-01159-x)
Supplement: Supplementary file 1 — Additional file 1. Additional tables and figures. [file 12951_2021_1159_MOESM1_ESM.docx]

**Supplementary Information**

**A Core-Shell Au@Cu_2-x_Se Heterogeneous Metal Nanocomposite for Photoacoustic and Computed Tomography Dual-Imaging-Guided Photothermal Boosted Chemodynamic Therapy**

Le Zhang^1,3,4^, Chunjuan Jiang^1^, Bing Li^2,3,4^, Zhengwang Liu^1,3,4^, Bingxin Gu^1^, Simin He^1^, Panli Li^1^, Yun Sun^1,2,3,4*^, Shaoli Song^1,3,4*^

1. Department of Nuclear Medicine, Shanghai Proton and Heavy Ion Center, Fudan University Cancer Hospital, Shanghai 201321, China.

2. Department of Research and Development, Shanghai Proton and Heavy Ion Center, Shanghai 201321, China.

3. Shanghai Key Laboratory of Radiation Oncology (20dz2261000), Shanghai, China.

4. Shanghai Engineering Research Center of Proton and Heavy Ion Radiation Therapy, Shanghai, China.

***Corresponding authors**

Yun Sun (yun.sun@sphic.org.cn)

Shaoli Song (shaoli-song@163.com)


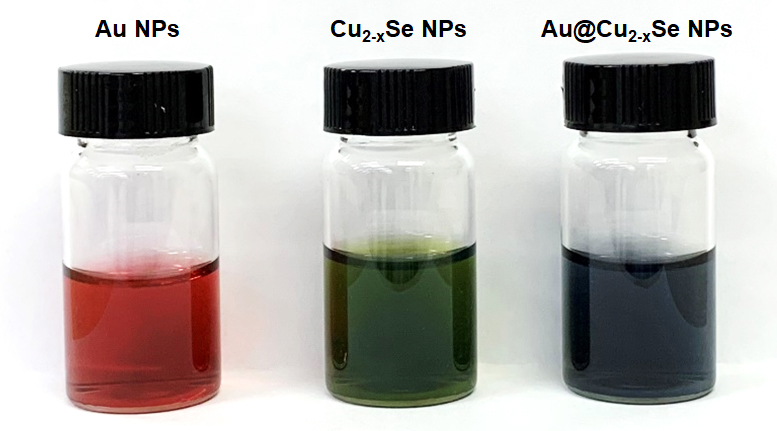


**Fig. S1** Photograph of Au NPs, Cu_2-x_Se NPs, and Au@Cu_2-x_Se NPs dispersed in H_2_O.


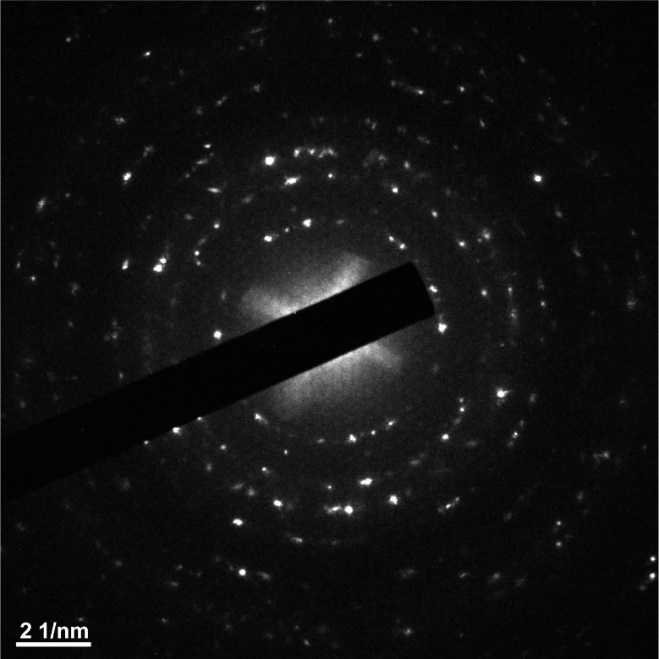


**Fig. S2** SAED pattern of Au@Cu_2-x_Se NPs.


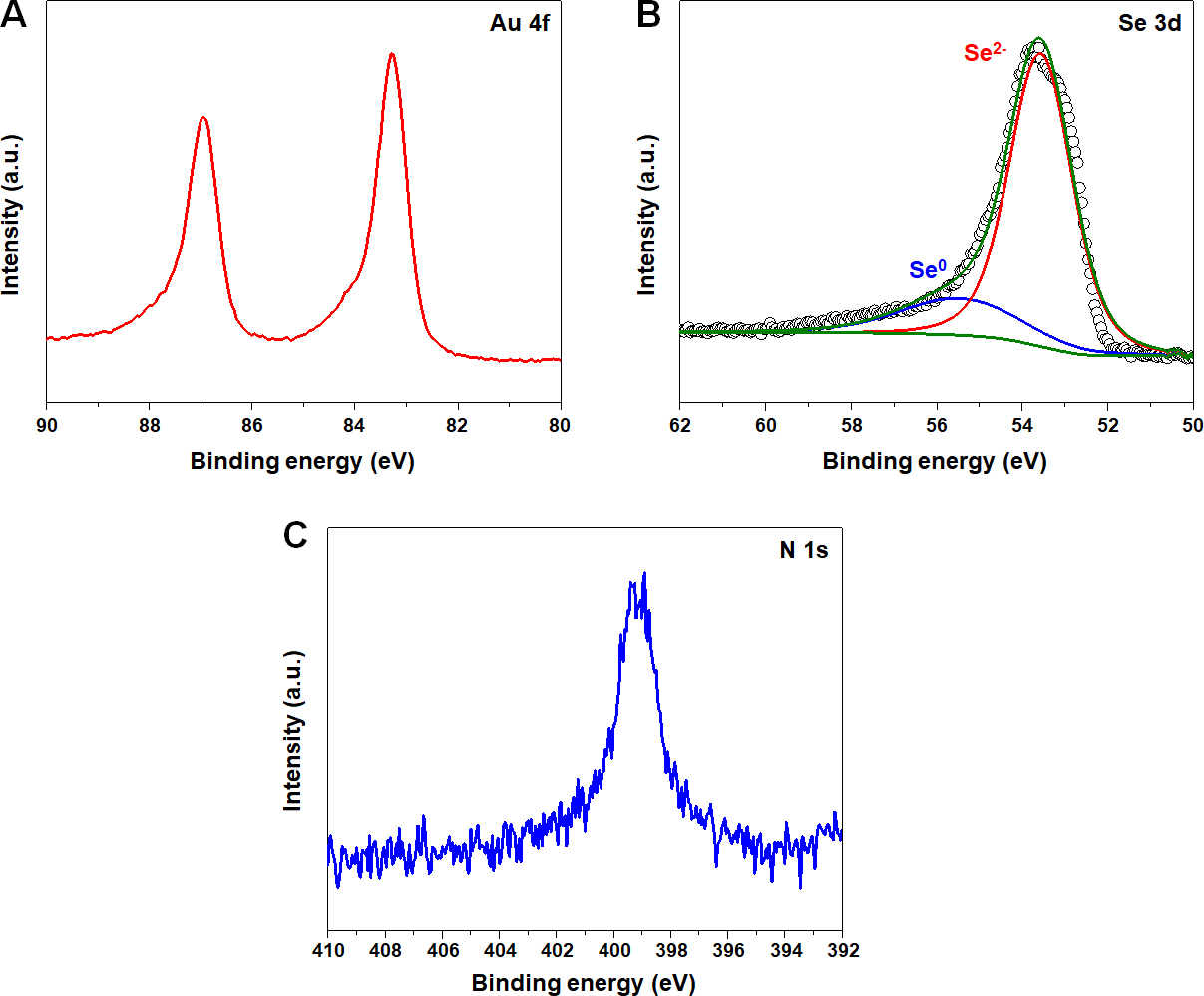


**Fig. S3** **A** Au 4f, **B** Se 3d, and **C** N 1s XPS spectrum of Au@Cu_2-x_Se NPs.


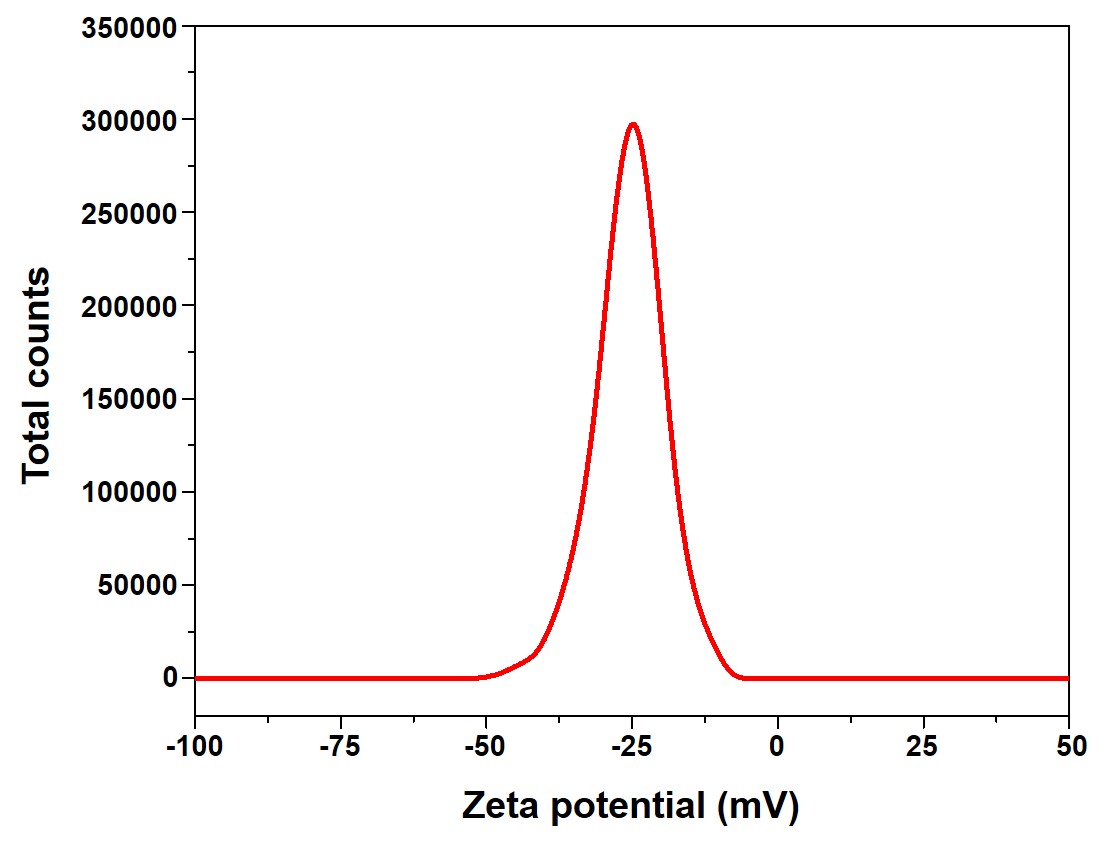


**Fig. S4** Zeta potential of Au@Cu_2-x_Se NPs dispersed in water (100 μg mL^-1^).


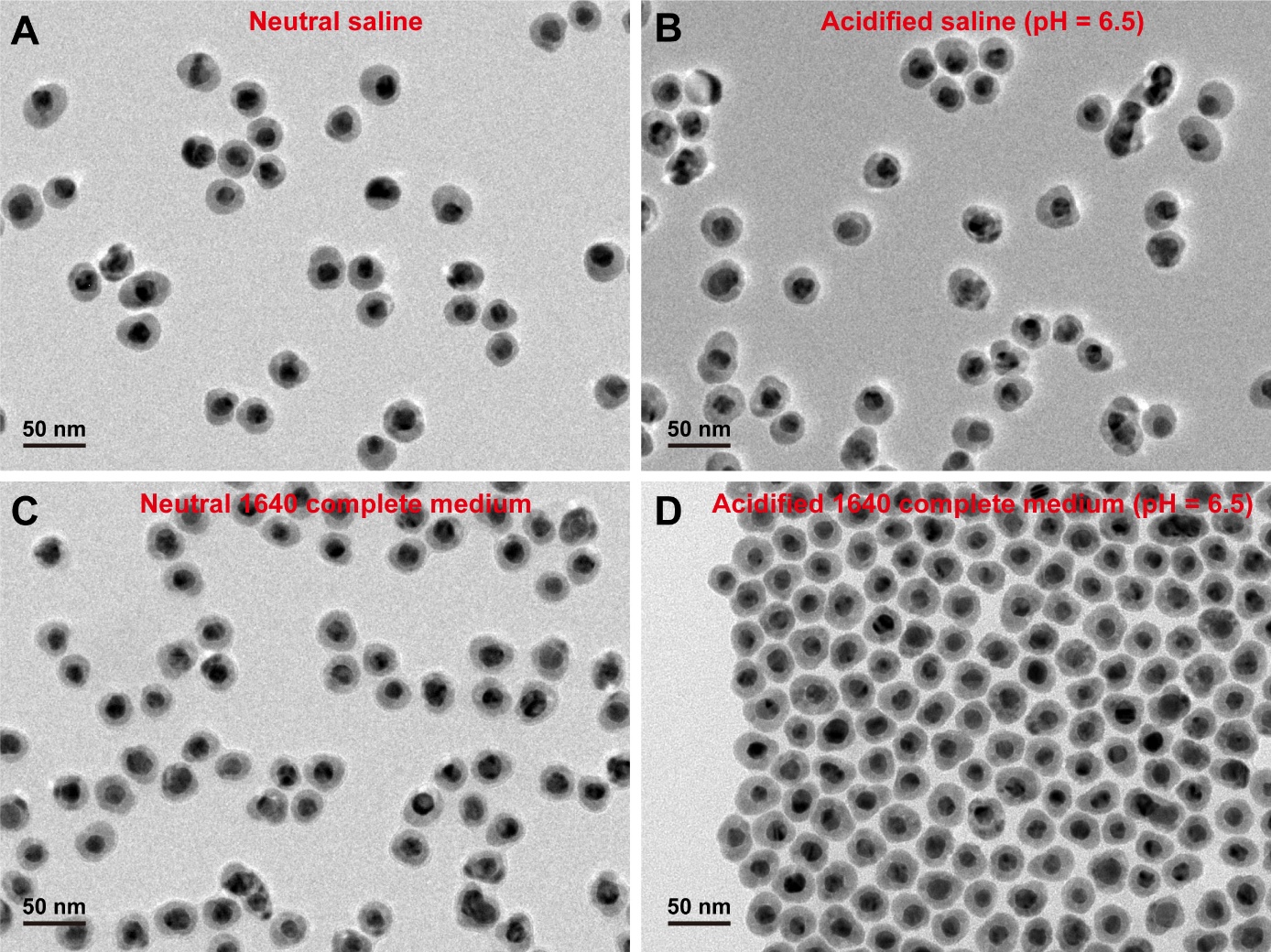


**Fig. S5** TEM images of Au@Cu_2-x_Se NPs after 14-day incubations in **A** neutral saline, **B** acidified saline (pH = 6.5), **C** neutral RPMI-1640 complete medium, and **D** acidified RPMI-1640 complete medium (pH = 6.5), respectively.

**Note:** Because tumor microenvironment (TME) is weakly acid, the morphology changes of Au@Cu_2-x_Se NPs in TME simulation condition were observed. The pH of saline or RPMI-1640 was adjusted by diluted HCl. The morphology of Au@Cu_2-x_Se NPs after 14-day incubation both in neutral and acidic condition have no obvious changes (Fig. S5), indicating the excellent stability of Au@Cu_2-x_Se NPs.


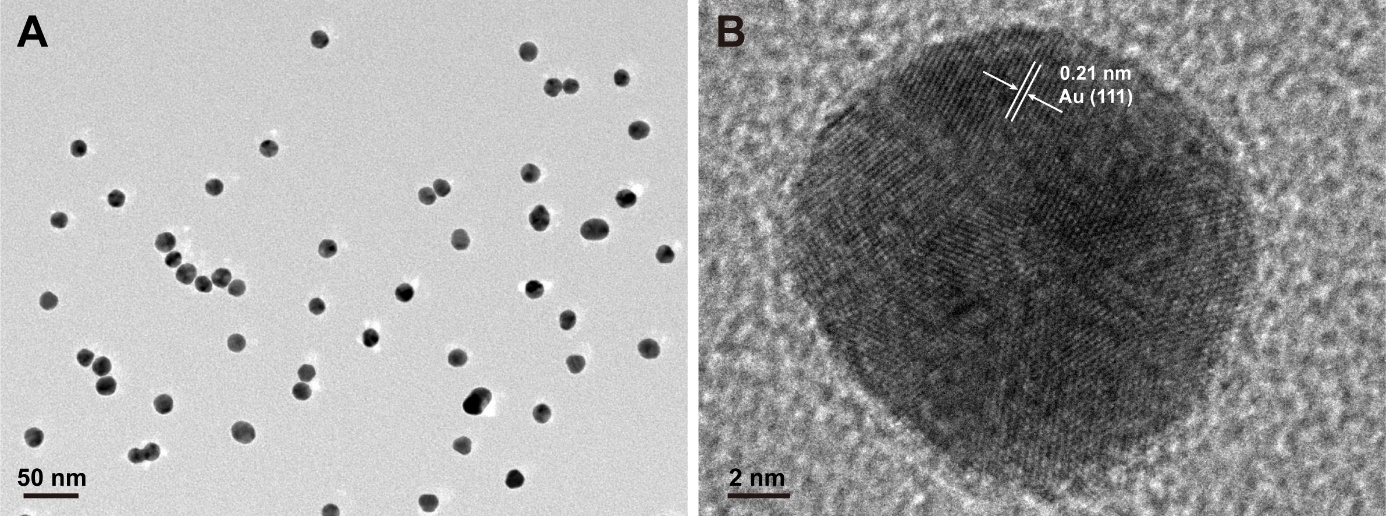


**Fig. S6** **A** TEM and **B** HRTEM images of Au NPs.


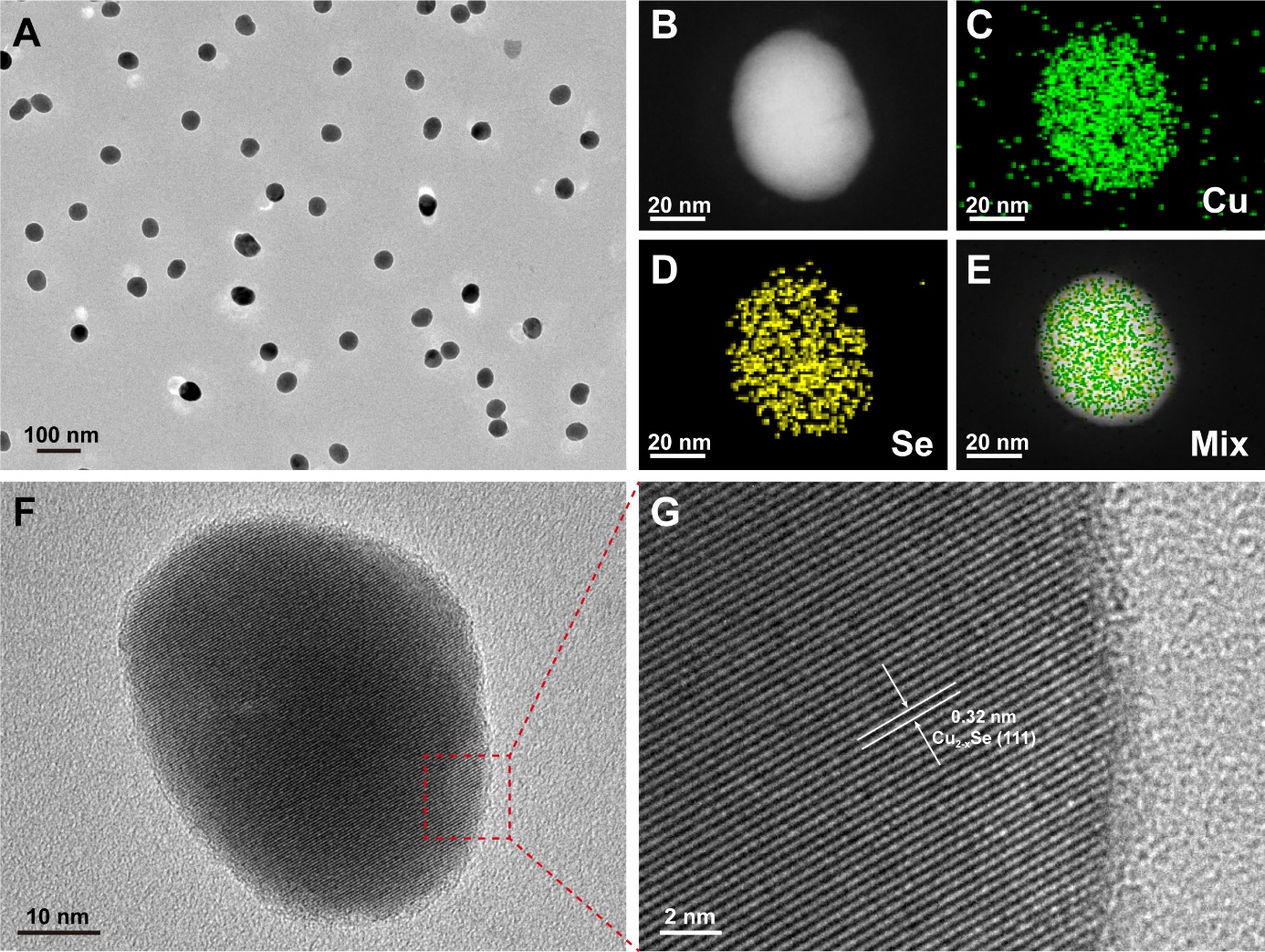


**Fig. S7** **A** TEM image, **B-E** elemental mapping images, and **F**, **G** HRTEM images of Cu_2-x_Se NPs.


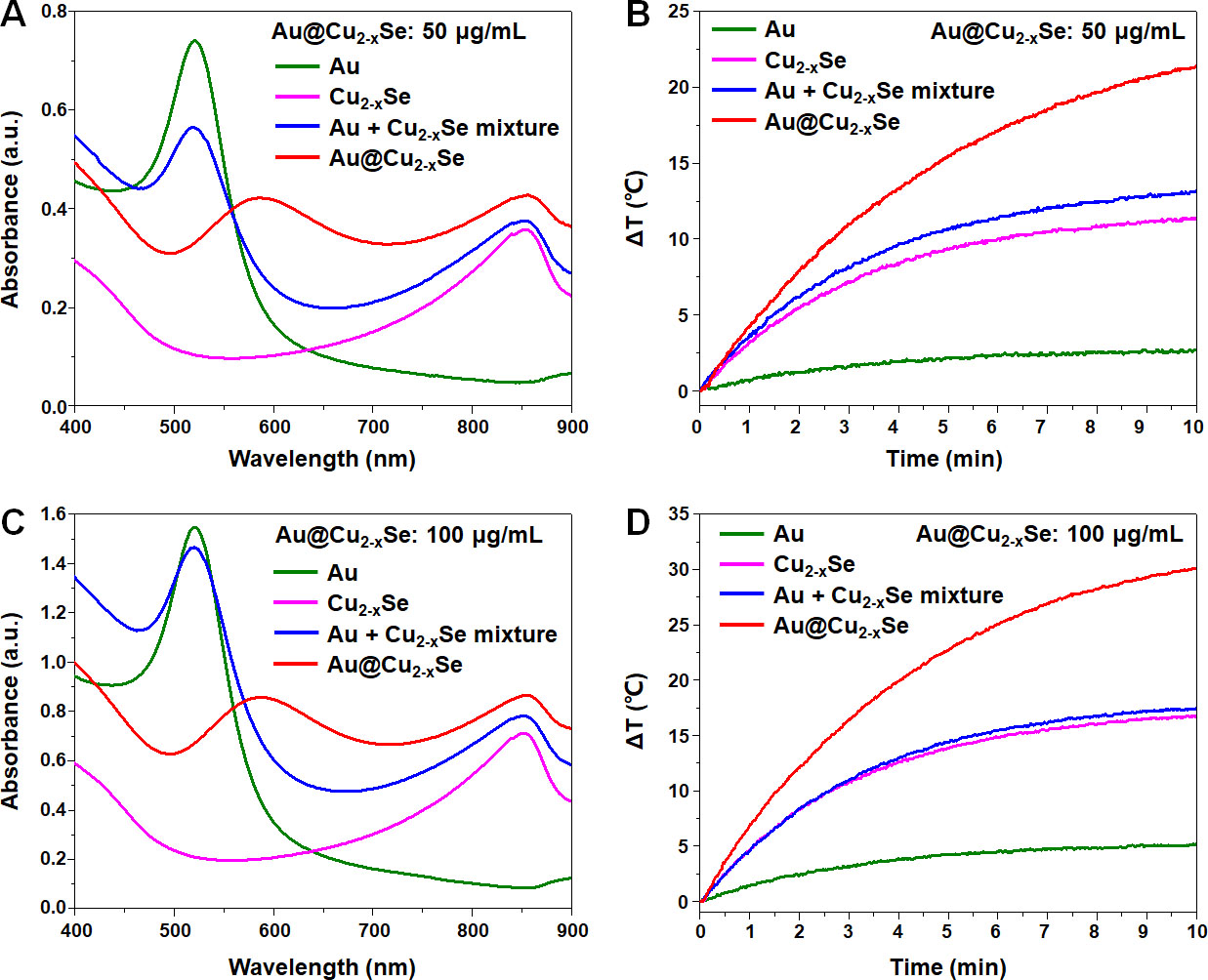


**Fig. S8** **A** UV-vis-NIR absorption spectra and **B** photothermal curves for Au NPs (21 μg mL^-1^), Cu_2-x_Se NPs (29 μg mL^-1^), Au@Cu_2-x_Se NPs (50 μg mL^-1^), and physical mixture of Au NPs and Cu_2-x_Se NPs aqueous suspensions. **C** UV-vis-NIR absorption spectra and **D** photothermal curves for Au NPs (42 μg mL^-1^), Cu_2-x_Se NPs (58 μg mL^-1^), Au@Cu_2-x_Se NPs (100 μg mL^-1^), and physical mixture of Au NPs and Cu_2-x_Se NPs aqueous suspensions. The power density of 808 nm NIR was maintained at 1.0 W cm^-2^ in photothermal tests.

**Note:** The mass ratios of Au and Cu_2-x_Se in Au@Cu_2-x_Se NPs determined by ICP-OES were 42.3 wt% and 57.7 wt%, respectively. To ensure that the photothermal performance of Au NPs, Cu_2-x_Se NPs, and Au@Cu_2-x_Se NPs were compared under the same conditions, the concentrations for Au@Cu_2-x_Se NPs, Au NPs, and Cu_2-x_Se NPs aqueous suspensions in Fig. S8 were set at 50, 21, and 29 μg mL^-1^, or 100, 42, and 58 μg mL^-1^, respectively.


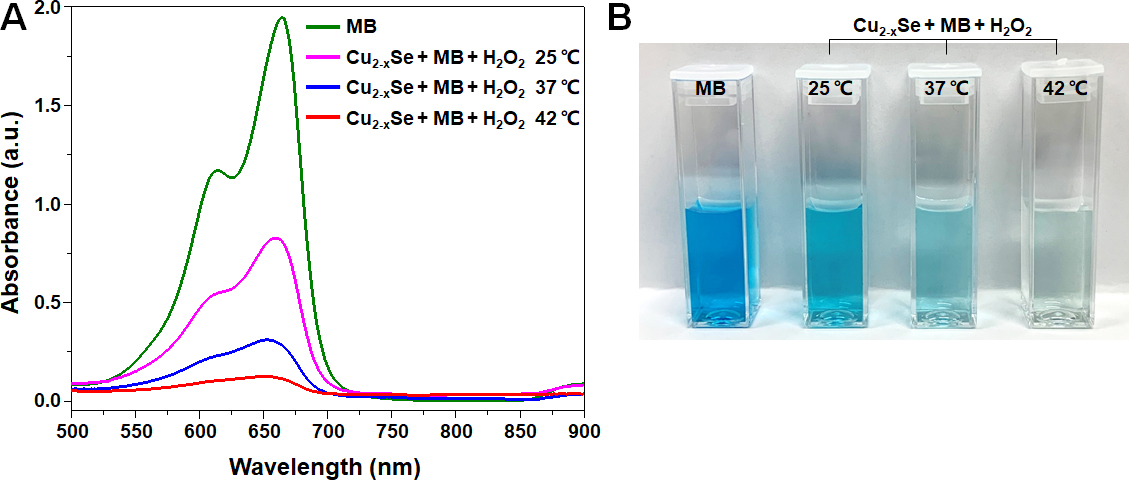


**Fig. S9 A** UV-vis-NIR absorption spectra of MB aqueous solutions containing Cu_2-x_Se NPs and H_2_O_2_ after treatments at different temperatures. **B** Photograph shows the corresponding color changes of MB solutions. Final concentrations of Cu_2-x_Se NPs, MB, and H_2_O_2_ were set at 58 μg mL^-1^, 10 μg mL^-1^, and 10 mM, respectively.

**Note:** The mass ratios of Au and Cu_2-x_Se in Au@Cu_2-x_Se NPs determined by ICP-OES were 42.3 wt% and 57.7 wt%, respectively. The concentration of Au@Cu_2-x_Se used in MB degradation assay was 100 μg mL^-1^ (Fig. 3A). So the concentration of Cu_2-x_Se used in MB degradation assay was set at 58 μg mL^-1^ (Fig. S9).


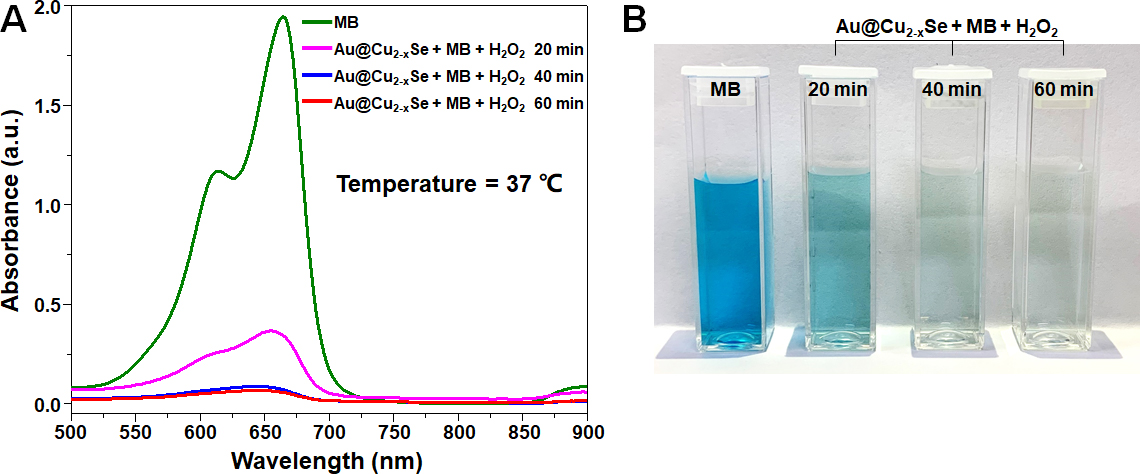


**Fig. S10** **A** UV-vis-NIR absorption spectra of MB aqueous solutions containing Au@Cu_2-x_Se NPs and H_2_O_2_ after treatments at 37 °C for 20, 40, and 60 min, respectively. **B** Photograph shows the corresponding color changes of MB solutions. Final concentrations of Au@Cu_2-x_Se, MB, and H_2_O_2_ were set at 100 μg mL^-1^, 10 μg mL^-1^, and 10 mM, respectively.


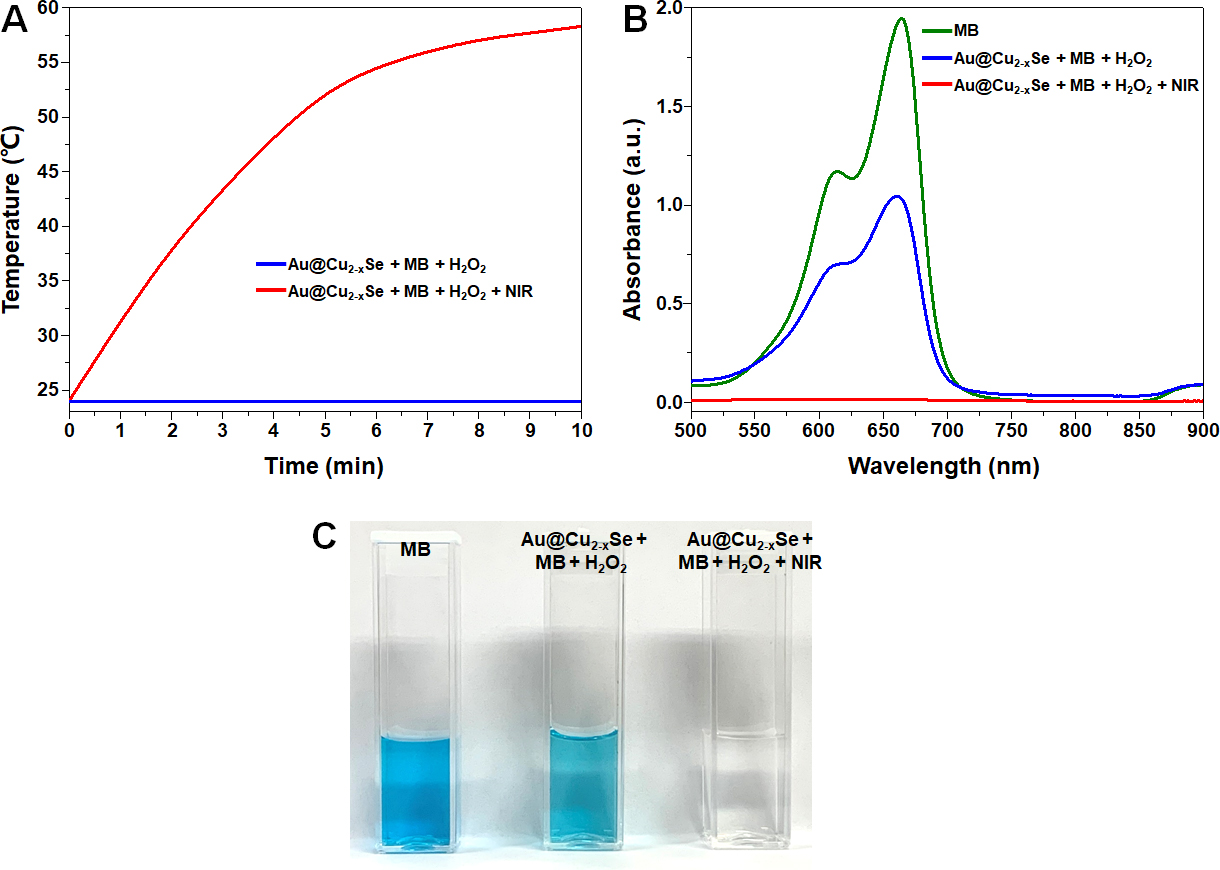


**Fig. S11** **A** Temperature rise curve of MB aqueous solution containing Au@Cu_2-x_Se NPs and H_2_O_2_ under NIR irradiation. **B** UV-vis-NIR absorption spectra and **C** corresponding color changes of MB aqueous solution after removing Au@Cu_2-x_Se NPs in A. Au@Cu_2-x_Se: 100 μg mL^-1^. MB: 10 μg mL^-1^. H_2_O_2_: 10 mM. NIR: 808 nm laser, 1.0 W cm^-2^ for 10 min.


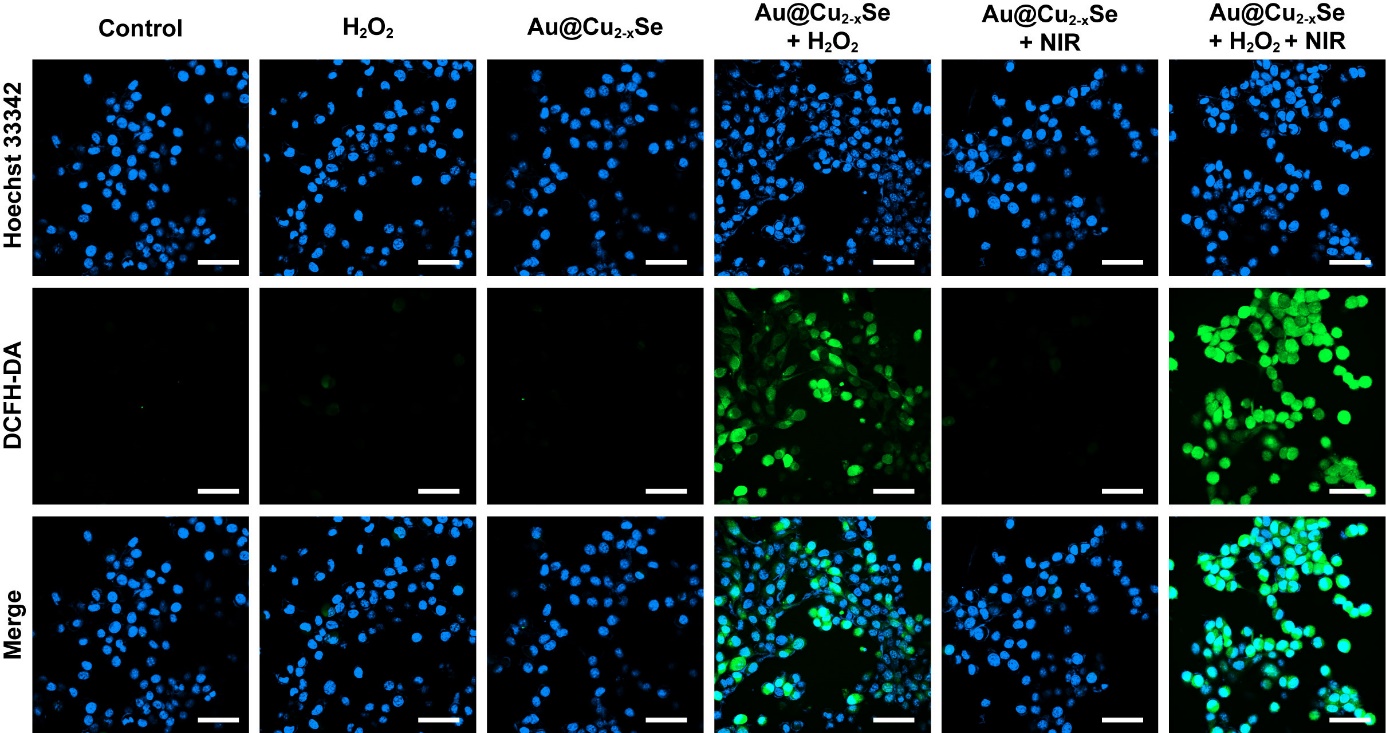


**Fig. S12** CLSM images of 4T1 tumor cells after different treatments, where Hoechst 33342 (blue) and DCFH-DA (green) were used to observe the cell nucleus and ROS generation, respectively. Scale bar = 50 μm. Au@Cu_2-x_Se: 50 μg mL^-1^. H_2_O_2_: 100 μM. NIR: 808 nm laser, 1.0 W cm^-2^ for 5 min.


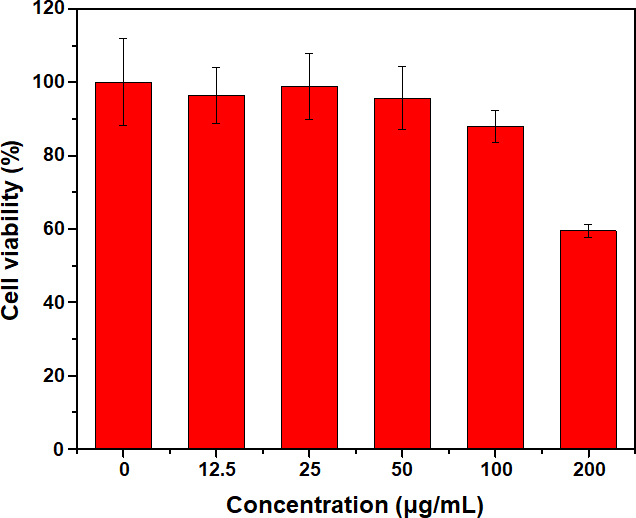


**Fig. S13** Cytotoxicity of Au@Cu_2-x_Se NPs toward HEK293 normal cells at different concentrations. Data are presented as mean ± s.d. (n = 6).


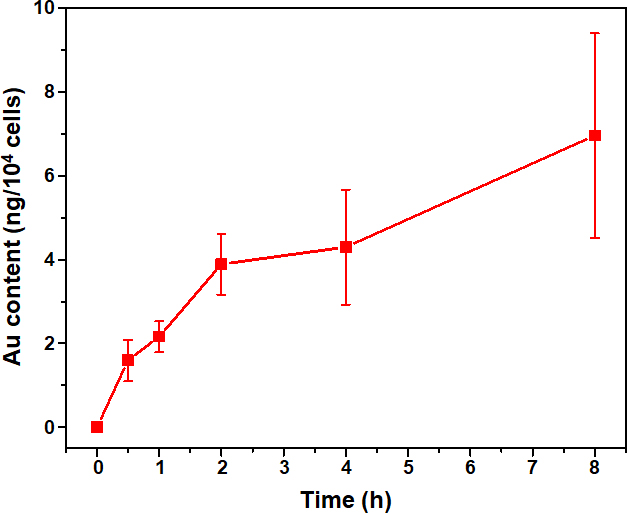


**Fig. S14** Time-dependent cellular uptake of Au@Cu_2-x_Se NPs measured by ICP-MS after incubation at the concentration of 50 μg mL^-1^. Data are presented as mean ± s.d. (n = 3).


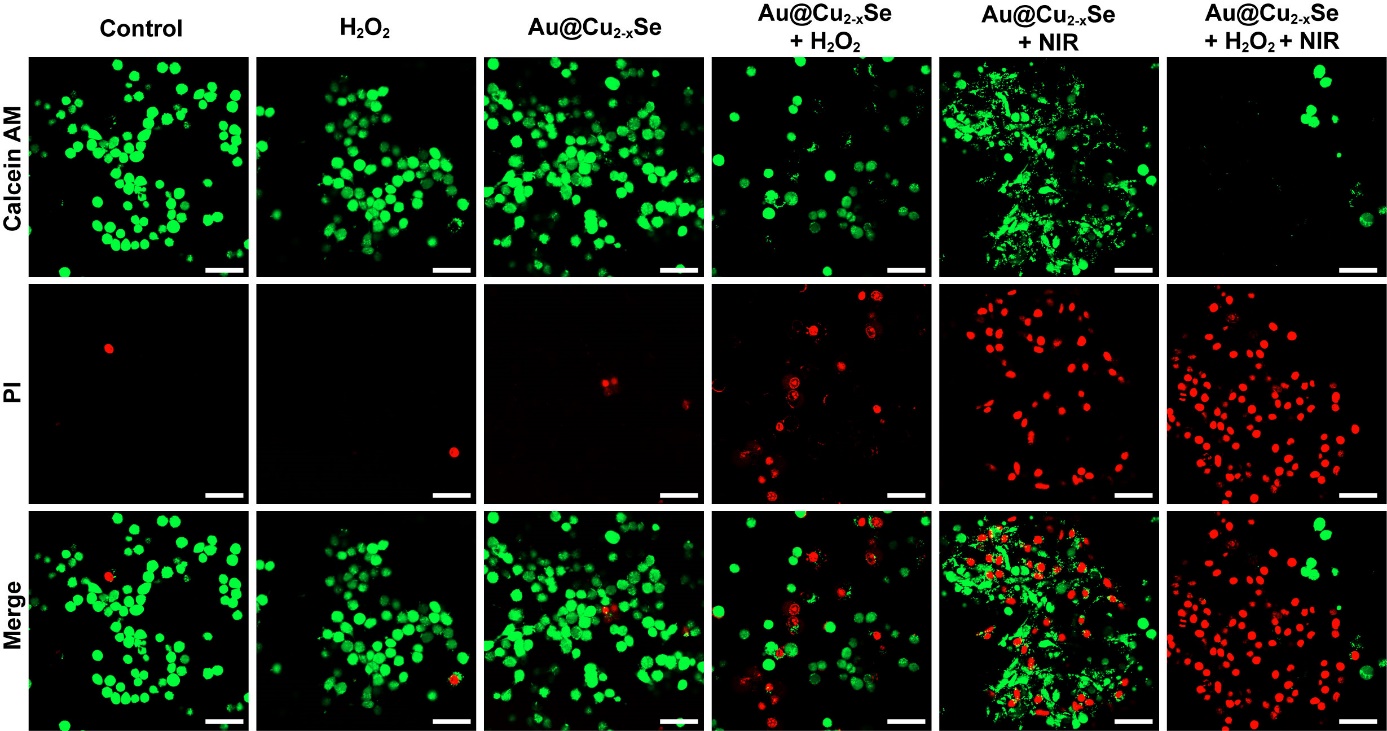


**Fig. S15** CLSM images of 4T1 tumor cells after different treatments, where Calcein AM (green) and PI (red) were used to observe the living and dying cells, respectively. Scale bar = 50 μm. Au@Cu_2-x_Se: 50 μg mL^-1^. H_2_O_2_: 100 μM. NIR: 808 nm laser, 1.0 W cm^-2^ for 5 min.


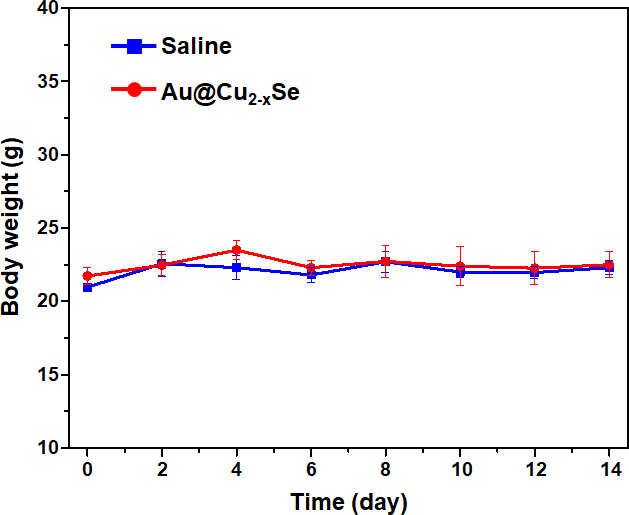


**Fig. S16** Mice body weight changes for *in vivo* toxicity evaluation after intravenous injection of 200 μL saline or 200 μL saline containing Au@Cu_2-x_Se NPs (2 mg mL^-1^). Data are presented as mean ± s.d. (n = 3).


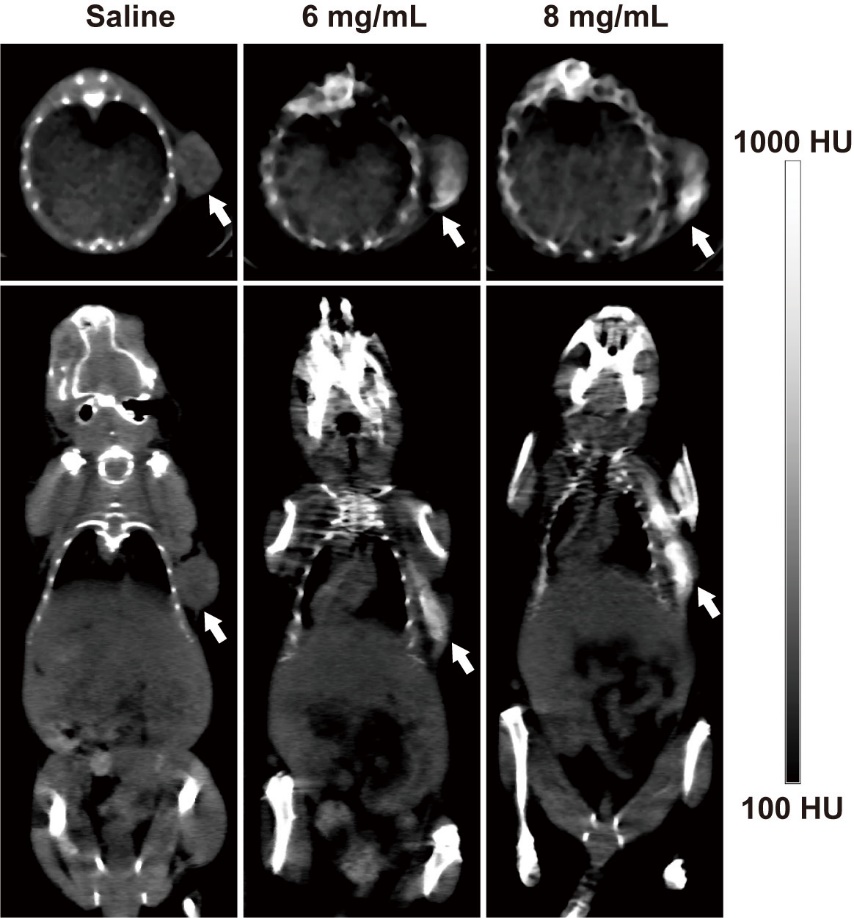


**Fig. S17** CT images of 4T1 tumor-bearing mice after intratumoral injections of 150 μL saline containing Au@Cu_2-x_Se NPs with 0, 6, and 8 mg mL^-1^, respectively. The tumor site is indicated by a white arrow.


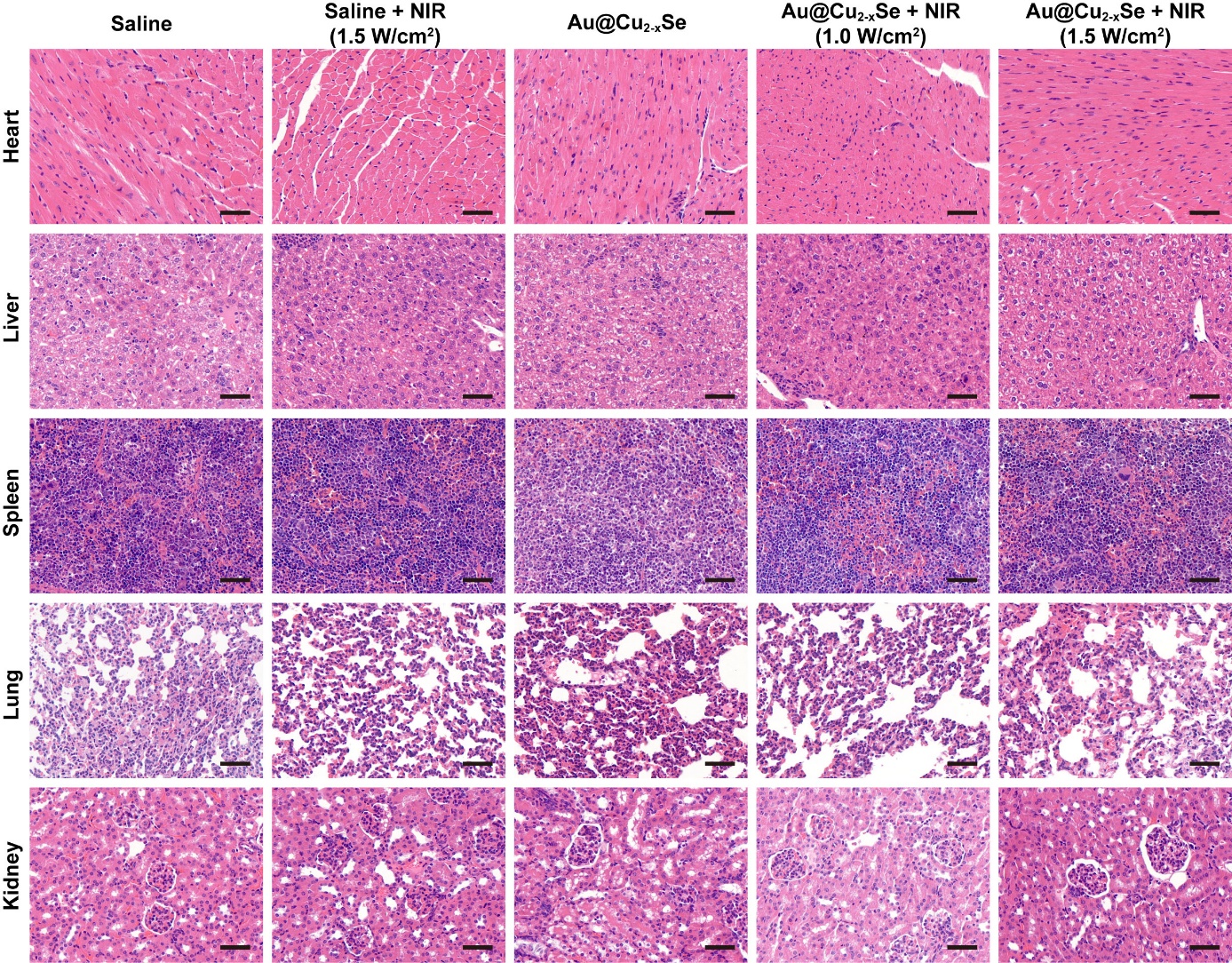


**Fig. S18** H&E staining on major organs from mice receiving different treatments at day 22. 4T1 tumor-bearing mice were treated by intravenous injection of 200 μL pure saline or 200 μL saline containing Au@Cu_2-x_Se NPs (2 mg mL^-1^) followed by 808 nm NIR irradiation at 1.0 or 1.5 W cm^-2^ for 5 min. Scale bar = 50 μm.


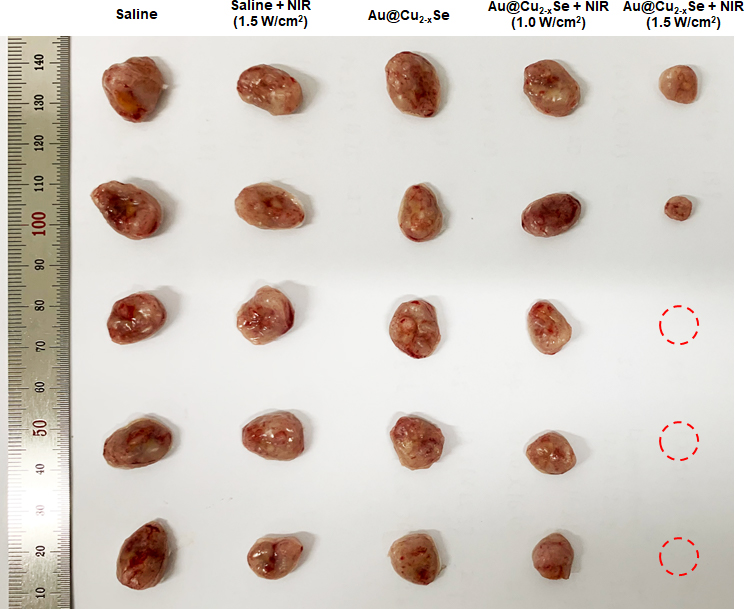


**Fig. S19** Photograph of tumors collected from mice receiving different treatments at day 22. 4T1 tumor-bearing mice were treated by intravenous injection of 200 μL pure saline or 200 μL saline containing Au@Cu_2-x_Se NPs (2 mg mL^-1^) followed by 808 nm NIR irradiation at 1.0 or 1.5 W cm^-2^ for 5 min.


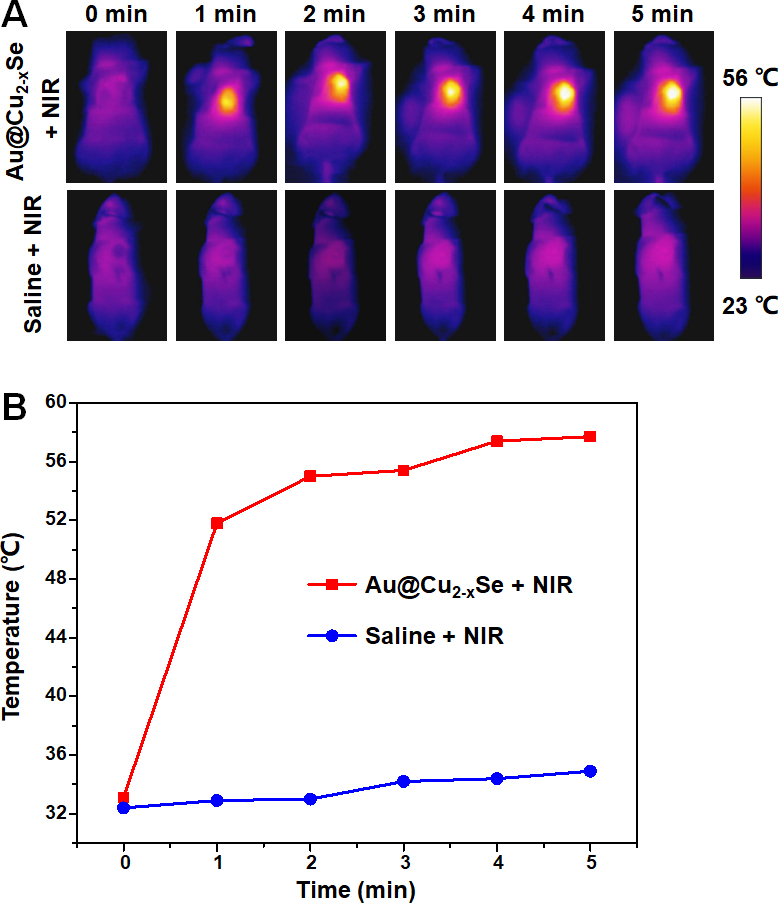


**Fig. S20 A** Thermal images and **B** temperature rise curves at tumor sites of 4T1 tumor-bearing mice after intratumoral injection of 100 μL pure saline or 100 μL saline containing Au@Cu_2-x_Se NPs (2 mg mL^-1^) followed by 808 nm NIR irradiation at 0.5 W cm^-2^ for 5 min.


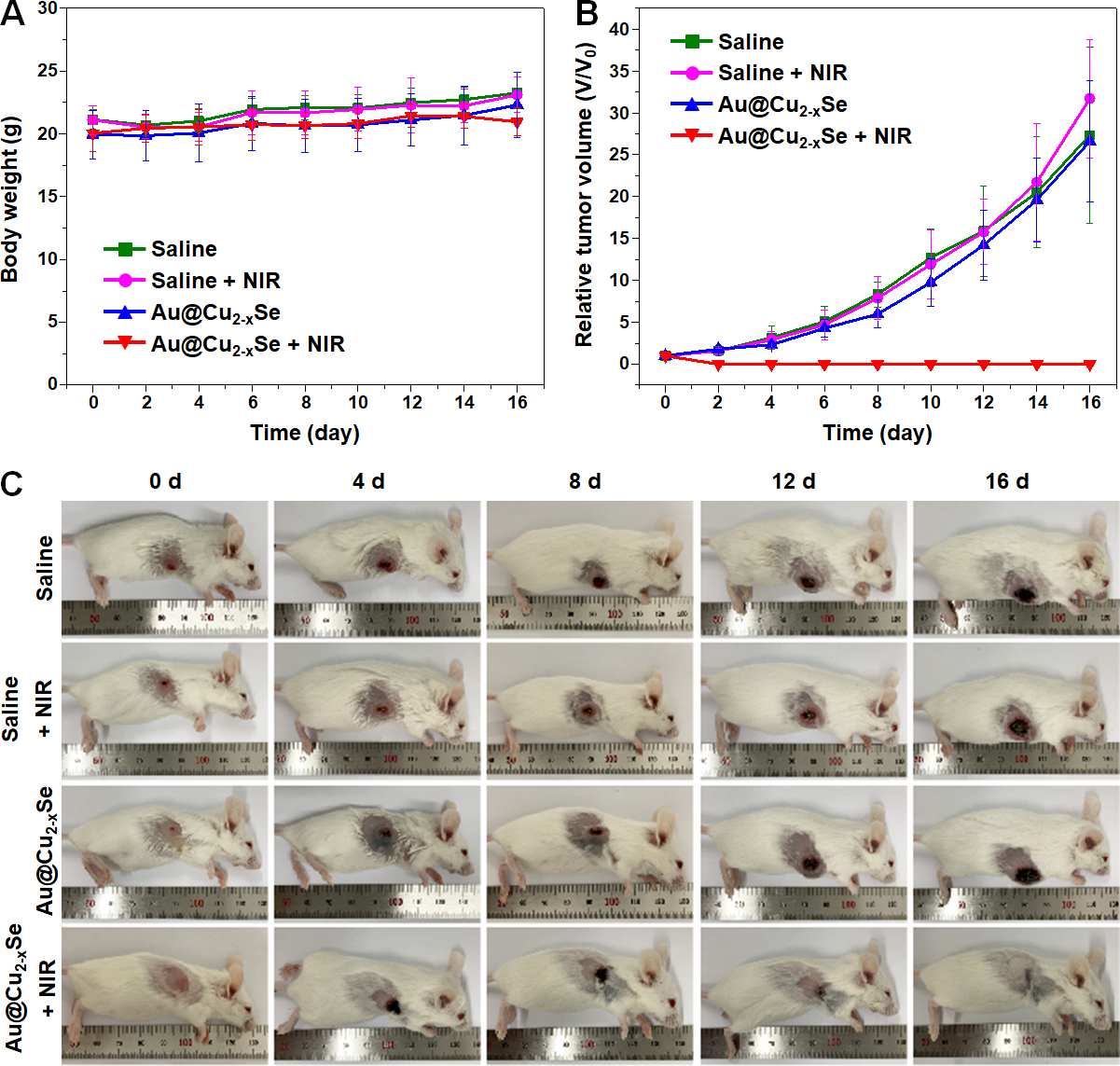


**Fig. S21 A** Body weight changes, **B** tumor volume growth curves, and **C** photographs of 4T1 tumor-bearing mice after intratumoral injection of 100 μL pure saline or 100 μL saline containing Au@Cu_2-x_Se NPs (2 mg mL^-1^) followed by 808 nm NIR irradiation at 0.5 W cm^-2^ for 5 min. Data are presented as mean ± s.d. (n = 5).


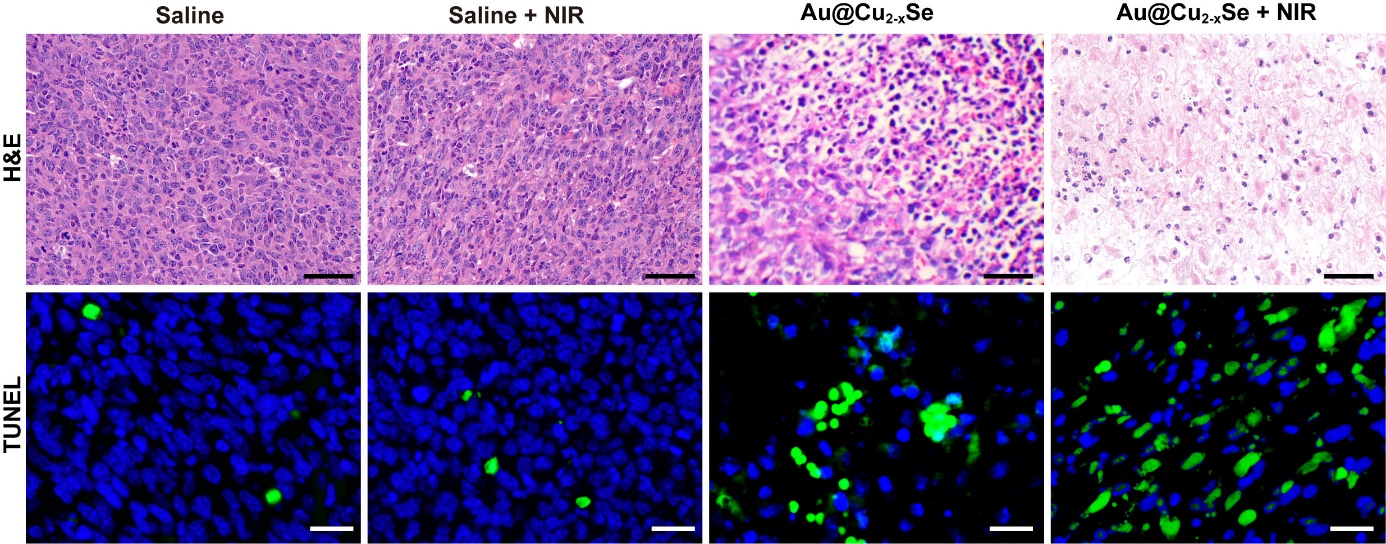


**Fig. S22** H&E and TUNEL immunofluorescence staining of tumor tissues at 20 h post-treatment. 4T1 tumor-bearing mice were treated by intratumoral injection of 100 μL pure saline or 100 μL saline containing Au@Cu_2-x_Se NPs (2 mg mL^-1^) followed by 808 nm NIR irradiation at 0.5 W cm^-2^ for 5 min. Scale bar = 50 μm.

**Table S1** Comparison of photothermal conversion efficiency (*η*) of recently reported photothermal agents.

| **Materials** | ***η* (%)** | **Wavelength (nm)** | **Reference** |
| --- | --- | --- | --- |
| Au@Cu_2-x_Se nanoparticles | 56.6 | 808 | This work |
| Bi/MnPcE_4_ nanocomposites | 33.7 | 808 | Biomaterials. 2020;228:119569 |
| V_2_C nanosheets | 48 | 808 | Angew Chem Int Ed. 2020;59:6601–6606 |
| Polypyrrole Nanoparticles | 33.35 | 808 | Adv Funct Mater. 2021;31:2008362 |
| Nb_2_C nanosheets | 36.4 | 808 | J Am Chem Soc. 2017;139:16235−16247 |
| Pd nanosheets | 30.9 | 808 | J Am Chem Soc. 2020;142:5649−5656 |
| PVP-Bi nanodots | 30 | 808 | Adv Funct Mater. 2017;27:1702018 |
| NbSe_2_ nanosheets | 42.9 | 808 | Adv Funct Mater. 2020;30:2001593 |
| Tungsten-based  polyoxometalate nanoclusters | 58 | 808 | ACS Nano. 2020;14:2126–2136 |
| Au-Fe_2_C Janus Nanoparticles | 30.2 | 808 | ACS Nano. 2017;11:9239−9248 |
| BSA-CuFeS_2_ nanoparticles | 38.8 | 808 | ACS Appl Mater Interfaces. 2019;11:18133−18144 |
| COF-CuSe nanoparticles | 26.34 | 808 | ACS Appl Mater Interfaces. 2019;11:23072−23082 |
| Au@Cu_2-x_S Nanocrystals | 59 | 808 | Adv Mater. 2016;28:3094–3101 |
